# The efficiency and safety of Shengxuening tablet on treating and preventing iron deficiency anemia: A systematic review and meta-analysis

**DOI:** 10.3389/fphar.2022.1029641

**Published:** 2022-11-03

**Authors:** Yanyu Zhang, Yan Lv, Yan Sun, Yumeng Li, Dehao Wang, Jicong Niu, Pei Zhao, Mei Zhang, Mingjing Wang, Weiyi Liu, Xiaomei Hu

**Affiliations:** ^1^ Department of Hematology, Xiyuan Hospital, China Academy of Chinese Medical Sciences, Beijing, China; ^2^ Graduate School, China Academy of Chinese Medical Sciences, Beijing, China; ^3^ Graduate School, Beijing University of Chinese Medicine, Beijing, China; ^4^ Department of Hematology, Beijing Longfu Hospital, Beijing, China

**Keywords:** Shengxuening tablet, iron deficiency anemia, meta-analysis, systematic review, random controlled trials

## Abstract

**Background:** Iron deficiency anemia (IDA) is a public health problem worldwide. Shengxuening Tablet (SXN) has been used for the treatment of various types of anemia, attaining high efficacy.

**Objective:** To evaluate the safety of SXN as well as its preventive and therapeutic efficacy against IDA across different population groups.

**Methods:** PubMed, Embase, Web of Science, the Cochrane Library, the China Knowledge Network, the China Biomedical Literature Database, the Wanfang Data Knowledge Service Platform, and the China Science and Technology Journal Database was searched for relevant clinical trials through June 2022 and a systematic review and meta-analysis of the identified studies was undertaken.

**Results:** A total of 39 trials involving 4,562 cases were included in the meta-analysis. The total efficiency of SXN was superior than the control group in improving red blood cell (RBC) count [SMD = 1.31, 95% CI (0.7, 1.91), *p* < 0.0001], hemoglobin (Hb) [SMD = 1.11, 95% CI (0.75, 1.46), *p* < 0.00001], mean corpuscular volume (MCV) [SMD = 0.5, 95% CI (0.33, 0.68), *p* < 0.00001], total serum iron (SI) levels [SMD = 1.87, 95% CI (1.3, 2.44), *p* < 0.00001], and transferrin saturation (TSAT) levels [SMD = 2.07, 95% CI (1.86, 2.27), *p* < 0.00001]. Besides, the total effects of SXN to improve mean corpuscular hemoglobin (MCH) [SMD = 0.12, 95% CI (−0.16, 0.4), *p* = 0.41], mean corpuscular hemoglobin concentration (MCHC) [SMD = 0.03, 95% CI (−0.18, 0.24), *p* = 0.77], hematocrit (HCT) [SMD = 0.65, 95% CI (−0.25, 1.55), *p* = 0.16], and serum ferritin (SF) levels [SMD = 0.59, 95% CI (−0.67, 1.85), *p* = 0.36] and reduce the total iron binding capacity (TIBC) [SMD = 0.34, 95% CI (−0.07, 0.74), *p* = 0.1] was comparable to that of iron supplementation. SXN significantly raised the total effective rates of IDA [risk ratio (RR) = 1.06, 95% CI (1.02, 1.09), *p* = 0.0005] and was associated with fewer adverse events [RR = 0.24, 95% CI (0.18, 0.31), *p* < 0.00001], fewer adverse pregnancy outcomes [RR = 0.34, 95% CI (0.2, 0.57), *p* < 0.0001], and lower anemia recurrence rates during pregnancy [RR = 0.29, 95% CI (0.1, 0.84), *p* = 0.02]. Regarding prevention, the effects of SXN to maintain the RBC count, Hb level and other IDA-related parameters were comparable to that of control group and SXN reduced the risk of IDA incidence during pregnancy.

**Conclusion:** SXN demonstrated promising efficacy in the treatment and prevention of IDA and outperformed routine iron formulations in terms of safety, thus rendering SXN a reliable treatment option for IDA.

**Systematic Review Registration:** https://www.crd.york.ac.uk/prospero/, identifier: CRD42022353247.

## Introduction

Iron deficiency anemia (IDA) is the most common form of anemia worldwide. IDA is characterized by hypochromia with reduced blood hemoglobin (Hb), mean corpuscular volume (MCV), and mean corpuscular hemoglobin (MCH), as well as disorders of iron status, including abnormal serum ferritin (SF) levels, serum iron (SI) levels, total iron binding capacity (TIBC), and levels of transferrin saturation (TSAT) (Lopez et al., 2016). Iron replacement therapy is the standard treatment for IDA. However, gastrointestinal adverse effects are common with oral iron supplementations, while intravenous iron preparations can cause hypersensitivity-type and infusion reactions (Snook et al., 2021), which can lead to discontinuation of treatment and affect clinical outcomes.

Shengxuening Tablet (SXN) is a Chinese patent medicine extracted from silkworm excrement (Yang, 2021). Its main components are chlorophyll derivatives and sodium iron chlorophyllin. SXN is widely used and has exhibited therapeutic efficacy in clinical trials for various anemias. SXN-assisting cyclosporin A therapy was reported to be effective at treating chronic aplastic anemia (Zhang et al., 2006). A systematic review showed that SXN administration was more effective and safer than oral iron supplementation in patients with renal anemia (Zeng et al., 2020). Additionally, a phase IV clinical trial in China demonstrated that SXN was efficacious and safe for both adults and children with IDA, with a total effective rate of 84.8% (Ding et al., 2019). Given the accumulating evidence, we performed a systematic review and meta-analysis of the effect and safety of SXN in patients with IDA.

## Materials and methods

### Search strategy

PubMed, Embase, Web of Science, the Cochrane Library, the China Knowledge Network (CNKI), the China Biomedical Literature Database (CBM), the Wanfang Data Knowledge Service Platform, and the China Science and Technology Journal Database (VIP) were searched for clinical trials through June 2022 using the following key words: Shengxuening AND (“Anemia, Iron Deficiency” OR “Iron-Deficiency Anemia” OR “Iron Deficiency Anemia” OR “Anemias, Iron-Deficiency” OR “Anemias, Iron Deficiency” OR “Iron-Deficiency Anemias” OR “Iron Deficiency Anemias”). The review protocol has been registered in the PROSPERO International Prospective Register of Systematic Reviews (registration number: CRD42022353247).

### Inclusion and exclusion criteria

The inclusion criteria were as follows: 1) The study was a randomized controlled trial conducted in patients with IDA or healthy individuals; 2) the treatment group took SXN alone or in combination with one type of iron supplementation, while the control group received one type of iron formulation, placebo, or no intervention; 3) sufficient data were clearly reported, including basic information for each group, number of cases, intervention treatment courses, doses, and outcomes; and 4) the study reported baseline and post-intervention results on IDA-related indicators, including RBC count, Hb, MCV, MCH, MCHC, HCT, SI, SF, TIBC, TSAT, or IDA effective rate according to Diagnostic and Therapeutic Criteria of Hematological Diseases. These are 1) clinical cure, which refers to the restoration of Hb, SI, and TIBC levels and the disappearance of clinical symptoms; 2) marked effectiveness, which refers to the degree of recovery from severe to mild levels of anemia with an improvement of more than 2 grades and a significant improvement in clinical symptoms; 3) general effectiveness, which refers to a 1-grade improvement in the degree of anemia accompanied by an improvement in clinical symptoms; and 4) ineffectiveness, where no improvement in the severity of anemia or clinical symptoms is observed.

The exclusion criteria were as follows: 1) The article involved a meta-analysis, review, animal experimentation, or other studies irrelevant to clinical trials; 2) cases complicated with severe internal disease; 3) SXN was used in the control group; 4) conference articles; and 5) graduation papers.

The meta-analysis was divided according to different population groups, including children (aged < 14 years) and pregnant women, which are two vulnerable populations that susceptible to IDA, and the other population group comprised adults aged ≥18 years. Each group was further divided into the following three subgroups based on the type of treatment intervention: subgroup 1, where patients in the experimental group received SXN alone and those in the control group received one type of iron formulation; subgroup 2, in which patients in the experimental group received SXN combined with one type of iron formulation while patients in the control group were administered the same iron formulation alone; and subgroup 3, where the experimental group received SXN and the control group received no treatment.

### Data extraction

Two independent reviewers scanned the titles and abstracts of articles to identify studies that met the inclusion criteria. The full texts of eligible studies were screened and assessed by the two reviewers and inconsistencies were resolved. A characteristic table was designed to collect information of the studies, including the name of the first author, the year of publication, the study design methods, the number of cases, the population of participants, the duration and type of each intervention, and clinical outcomes.

### Quality assessment

Two investigators assessed the quality of the retrieved studies using the Cochrane risk-of-bias tool. The following dimensions were evaluated: randomization, allocation concealment, blinding, incomplete outcome data, selective reporting, and other bias. Disagreements were resolved through discussion with a third reviewer.

### Analysis methods

Meta-analysis was performed using RevMan 5.4. Since IDA-related parameters was measured in a variety of ways across studies (i.e., venous blood, capillary blood, arterial blood), the Standard mean difference (SMD) with 95% confidence interval (CI) was estimated for continuous outcomes. Risk ratio (RR) with 95% CI was estimated for dichotomous variables. Heterogeneity among the studies was assessed by the *I* ^2^ statistic. A fixed-effects model was used when the heterogeneity among studies was slight (*p* > 0.05, *I* ^2^ < 50%), while a random-effects model was employed when *I* ^2^ > 50%. The potential source of any significant heterogeneity was explored by meta-regression and subgroup analysis. Publication bias was evaluated by Egger’s test and sensitivity analysis was used to judge the influence of the individual studies on the pooled effect size. Meta-regression analysis, Egger’s test, and sensitivity analysis were performed using Stata MP 16.0.

### Study characteristic

**FIGURE 1:**
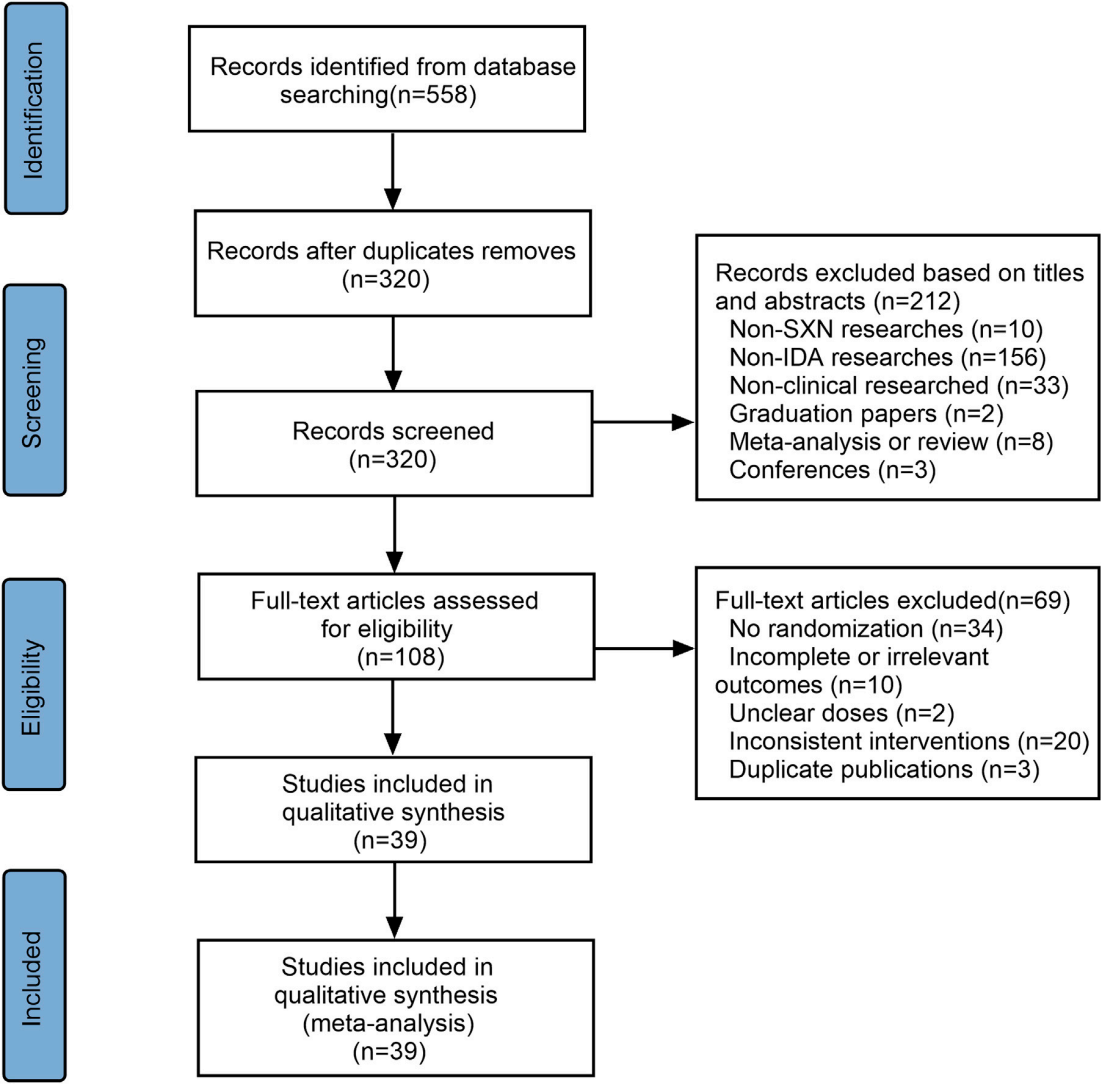
Flowchart showing the selection of trials for the meta-analysis and systematic review.

A total of 558 articles were initially selected—three from PubMed, five from Embase, two from Web of Science, three from the Cochrane Library, 271 from the CNKI, 80 from the CBM, 116 from the Wanfang Data Knowledge Service Platform, and 79 from the VIP database. Of these, 237 were duplicates and were excluded. Another 213 articles were excluded after reviewing the titles and abstracts as they did not meet the inclusion criteria. The remaining 108 references were assessed for eligibility and 69 were excluded due to insufficient data, no randomization, inconsistent outcomes, or duplication. A total of 39 articles were finally included (Figure 1), 10 of which were conducted in children <14 years of age, four involved adult populations, while remaining 25 were performed on pregnant women. The 10 trials involving children applied SXN in the treatment group and one iron formulation in the control group (subgroup 1). The four trials included adults all involved subgroup 1 therapy. In the pregnancy women-related trials, nine involved subgroup 1 treatment regimens, 14 involved subgroup 2 regimens, and two were in the subgroup 3 category. A total of 4,562 cases were included in these trials, 2,519 of which were allocated to experimental groups and 2,043 to control groups. The characteristics of the included studies are presented in Supplementary Table S1. Assessment methods for the various outcomes are presented in Supplementary Table S2. Among the selected studies, nine trials reported that IDA-related parameters were measured using venous blood samples, 11 reported that blood cell analyzers were applied for routine blood test. Automatic biochemistry analyzers were used in five trials while automatic immune analyzers were adopted in two trials for the evaluation of SI levels. Ferritin immunoelectron microscopies were used in five trials to assess TSAT levels. However, other trials did not mention the assessment methods for outcomes. A total of 10 kinds of oral iron formulations were involved in the 39 trials, and their main compositions were concluded in Supplementary Table S3.

### Literature quality

**FIGURE 2:**
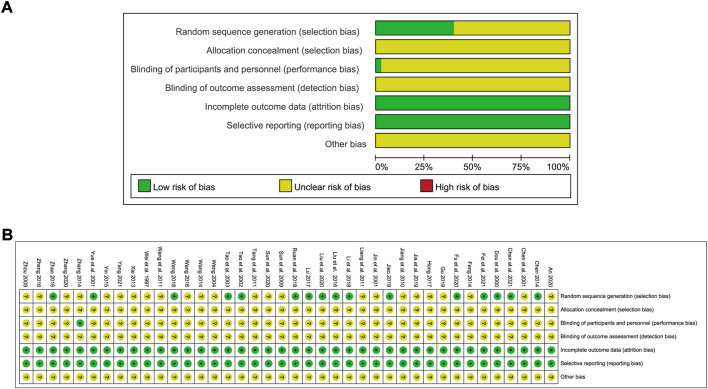
Risk of bias graph showing the quality of included studies. **(A)** Risk of bias graph; **(B)** Risk of bias summary.

Among the selected studies, 16 reported the random sequence generation method employed, one of which was a lottery while the others used random number tables. One study adopted a single-blind method while the rest did not report the blinding method employed. None of the studies described the method used for allocation concealment. The risk of bias graph is shown in Figure 2.

## Meta-analysis results of the therapeutic effect of Shengxuening tablet on iron deficiency anemia in different population groups

### Red blood cell count

**FIGURE 3:**
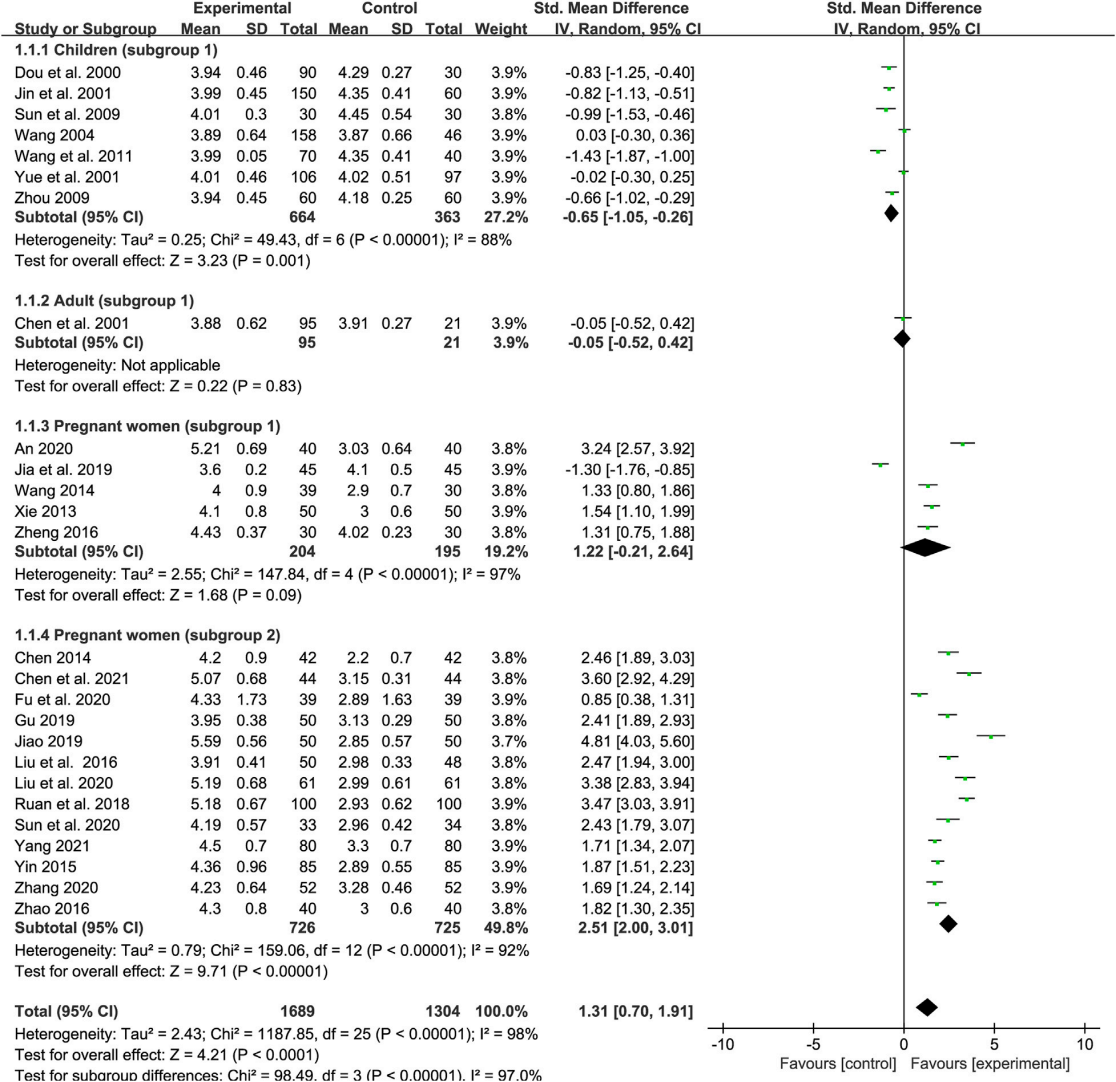
The forest plot assessing the effect of SXN on RBC counts.

We included 26 trials in our examination of the effect of SXN on the RBC count (seven studies included children, one trial included adults and other 18 trials included pregnant women). In total, 1,689 patients were assigned to the treatment group and 1,304 to the control group. A random-effects model was employed as the heterogeneity in total was high (*p* < 0.0001, *I* ^2^ = 98%). The results of the heterogeneity analysis across different populations were *p* < 0.00001, *I* ^2^ = 88% in children (subgroup 1); *p* < 0.00001, *I* ^2^ = 97% in pregnant women (subgroup 1); and *p* < 0.00001, *I* ^2^ = 92% in pregnant women (subgroup 2). Meta-regression analysis indicated that the heterogeneity in different population groups was not significantly related to the number of cases (*p* = 0.089 in children; *p* = 0.732 in subgroup 1 of pregnant women; and *p* = 0.719 in subgroup 2 of pregnant women), doses (*p* = 0.174 in children; *p* = 0.957 in subgroup 1 of pregnant women; and *p* = 0.732 in subgroup 2 of pregnant women), duration of treatment (*p* = 0.203 in subgroup 1 of pregnant women), or type of iron formulation (*p* = 0.101 in children, *p* = 0.496 in subgroup 1 of pregnant women; and *p* = 0.732 in subgroup 2 of pregnant women), and the effect size for different iron formulations were listed in Supplementary Table S4 when over two kinds of iron formulations were involved in the same population group. Sensitivity analysis showed that the results were stable and no single study affected the overall results when it was excluded (Figures 9A–C). Egger’s test indicated that no publication bias existed among the children (*p* = 0.083) and the subgroup 1 of pregnant women (*p* = 0.223), while there might be publication bias among the subgroup 2 of pregnant women (*p* = 0.025). The meta-analysis results are shown in Figure 3. The results showed that the total efficiency of SXN was superior than the control group [SMD = 1.31, 95% CI (0.7, 1.91), *Z* = 4.21 (*p* < 0.0001)]. Specifically, SXN was less effective compared with iron supplementations in children [SMD = −0.65, 95% CI (−1.05, −0.26), *Z* = 3.23 (*p* = 0.001)], and the efficacy of SXN in improving the RBC count was comparable to that of iron supplementations in adults [SMD = −0.05, 95% CI (−0.52, 0.42), *Z* = 0.22 (*p* = 0.83)] and pregnant women [SMD = 1.22, 95% CI (−0.21, 2.64), *Z* = 1.68 (*p* = 0.09)], while further analysis suggested that combined SXN/iron formulation treatment had a superior effect on this parameter when compared with iron formulation monotherapy in pregnant women [SMD = 2.51, 95% CI (2, 3.01), *Z* = 9.71 (*p* < 0.00001)].

### Hemoglobin

**FIGURE 4:**
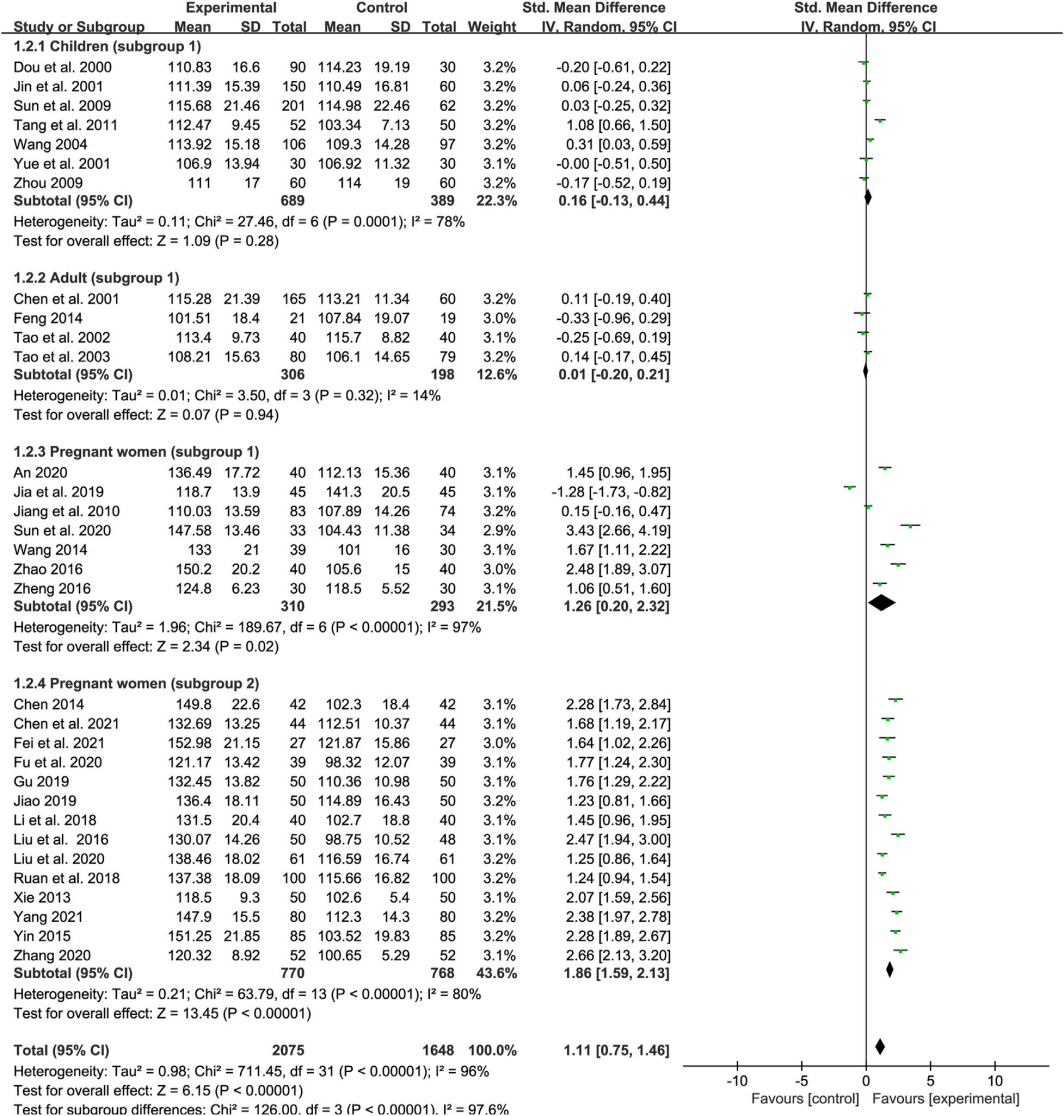
The forest plot assessing the effect of SXN on Hb levels.

A total of 32 studies examined the effect of SXN on Hb levels in IDA patients, of which seven trials involving children (subgroup 1), four trials involving adults (subgroup 1), seven trials involving pregnant women (subgroup 1), and 14 involving pregnant women (subgroup 2). The number of patients in the treatment and control groups was 2,075 and 1, 648, respectively. Heterogeneity differed among the groups (*p* < 0.00001, *I* ^2^ = 96% in total; *p* = 0.0001, *I* ^2^ = 78% in subgroup 1 of children; *p* = 0.32, *I* ^2^ = 14% in subgroup 1 of adults; *p* < 0.00001, *I* ^2^ = 97% in subgroup 1 of pregnant women; and *p* < 0.00001, *I* ^2^ = 80% in subgroup 2 of pregnant women). The heterogeneity was high among the studies and thus a random-effects model was applied. Meta-regression analysis showed that the heterogeneity in different population groups was not significantly related to the number of cases [*p* = 0.793 in children; *p* = 0.246 in adults; *p* = 0.294 in pregnant women (subgroup 1); and *p* = 0.719 in pregnant women (subgroup 2)], doses [*p* = 0.774 in children; *p* = 0.629 in pregnant women (subgroup 1); and *p* = 0.476 in pregnant women (subgroup 2)], and duration of treatment (*p* = 0.14 in subgroup 1 of pregnant women), but might related to the type of iron formulations in pregnant women (subgroup 2) (*p* = 0.019). The results showed that SXN had superior effect on boosting Hb levels compared with iron supplementation [SMD = 1.11, 95% CI (0.75, 1.46), *Z* = 6.15 (*p* < 0.00001)]. The efficacy of SXN in improving Hb levels was comparable to that of iron supplementation in children [SMD = 0.16, 95% CI (−0.13, 0.44), *Z* = 1.09 (*p* = 0.28)] and adults [SMD = 0.01, 95% CI (−0.2, 0.21), *Z* = 0.07 (*p* = 0.94)]. Moreover, the efficacy of SXN was superior in pregnant women notably [SMD = 1.26, 95% CI (0.2, 2.32), *Z* = 2.34 (*p* = 0.02) in subgroup 1; SMD = 1.86, 95% CI (1.59, 2.13), *Z* = 13.45 (*p* < 0.00001) in subgroup 2] (Figure 4). We further subdivided subgroup 2 of pregnant women into six groups according to the administered iron formulation for to further identify the effect of SXN on Hb, the data showed that SXN treatment in combination with an iron supplement all exhibited superior efficacy in increasing Hb levels when compared with different iron supplementation monotherapies, and the differences were all statistically significant (Supplementary Table S4). Sensitivity analysis indicated that the total effect size in different population groups were stable (Figures 9D–G) and no publication bias was observed based on Egger’s test in each population group [*p* = 0.872 in children; *p* = 0.071 in adults; *p* = 0.076 in pregnant women (subgroup 1); and *p* = 0.093 in pregnant women (subgroup 2)].

### Mean corpuscular volume

**FIGURE 5:**
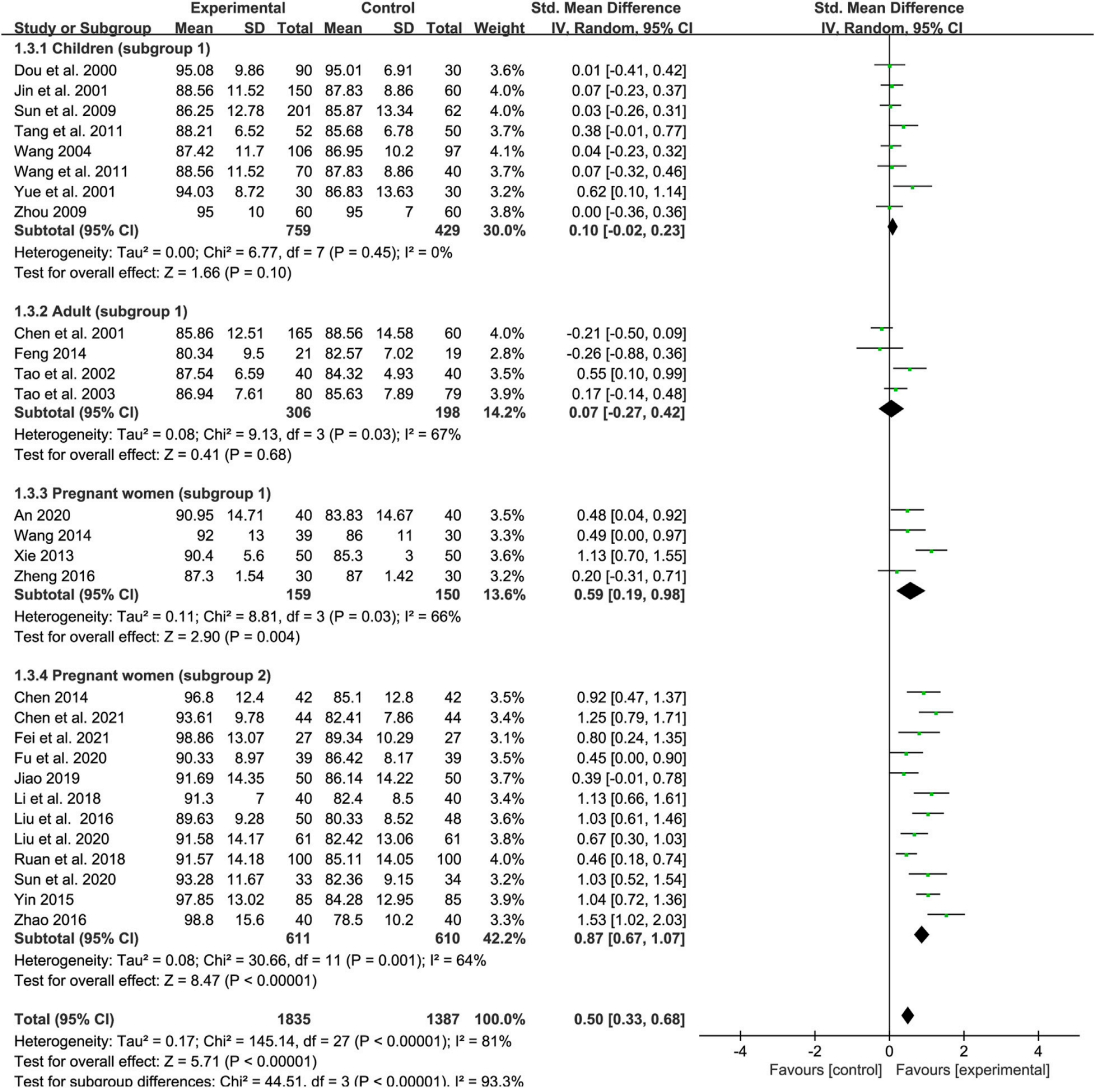
The forest plot assessing the effect of SXN on MCV levels.

A total of 28 trials reported data for MCV (eight for subgroup 1 of children, four for subgroup 1 of adults, four for subgroup 1 of pregnant women and 12 for subgroup 2 of pregnant women). A total of 1835 and 1,387 participants were enrolled in the treatment group and control group, respectively. The results of the heterogeneity analysis across different populations were *p* < 0.00001, *I* ^2^ = 81% in total; *p* = 0.45, *I* ^2^ = 0% in children (subgroup 1); *p* = 0.03, *I* ^2^ = 67% in adults (subgroup 1); *p* = 0.03, *I* ^2^ = 66% pregnant women (subgroup 1); and *p* = 0.001, *I* ^2^ = 64% in pregnant women (subgroup 2). A random-effects model was adopted due to the high heterogeneity among the studies. Meta-regression analysis showed that the heterogeneity in different population groups was not significantly related to the number of cases [*p* = 0.2 in children; *p* = 0.688 in adults; *p* = 0.103 in pregnant women (subgroup 1); and *p* = 0.285 in pregnant women (subgroup 1)], doses [*p* = 0.379 in children; and *p* = 0.592 in pregnant women (subgroup 1)], and duration of treatment [*p* = 0.379 in pregnant women (subgroup 1)], but might related to type of iron formulation in subgroup 2 of pregnant women (*p* = 0.048). Egger’s test showed no publication bias among the studies (*p* = 0.066; *p* = 0.841; *p* = 0.135; and *p* = 0.096) while sensitivity analysis suggested that the results were steady across different population groups (Figures 9H–K). The meta-analysis results are shown in Figure 5 and indicate that, overall, SXN showed greater efficacy in improving MCV levels compared with iron supplementation [SMD = 0.5, 95% CI (0.33, 0.68), *Z* = 5.71 (*p* < 0.00001)]. The efficacy of SXN in improving MCV levels was comparable to that of iron supplementation in children [SMD = 0.1, 95% CI (−0.02, 0.23), *Z* = 1.66 (*p* = 0.1)] and adults [SMD = 0.07, 95% CI (−0.27, 0.42), *Z* = 0.41 (*p* = 0.68)], while either SXN treatment alone or in combination with an iron supplement exhibited superior efficacy in increasing MCV levels when compared with iron supplementation monotherapy in pregnant women [SMD = 0.59, 95% CI (0.19, 0.98), *Z* = 2.9 (*p* = 0.004) in subgroup 1; SMD = 0.87, 95% CI (0.67, 1.07), *Z* = 8.47 (*p* < 0.00001) in subgroup 2]. Further analysis based on different iron formulation subgroups in pregnant women (subgroup 2) indicated that the effect of SXN treatment on MCV levels was not significantly affected by the type of iron formulations (Supplementary Table S4).

### Mean corpuscular hemoglobin

**FIGURE 6:**
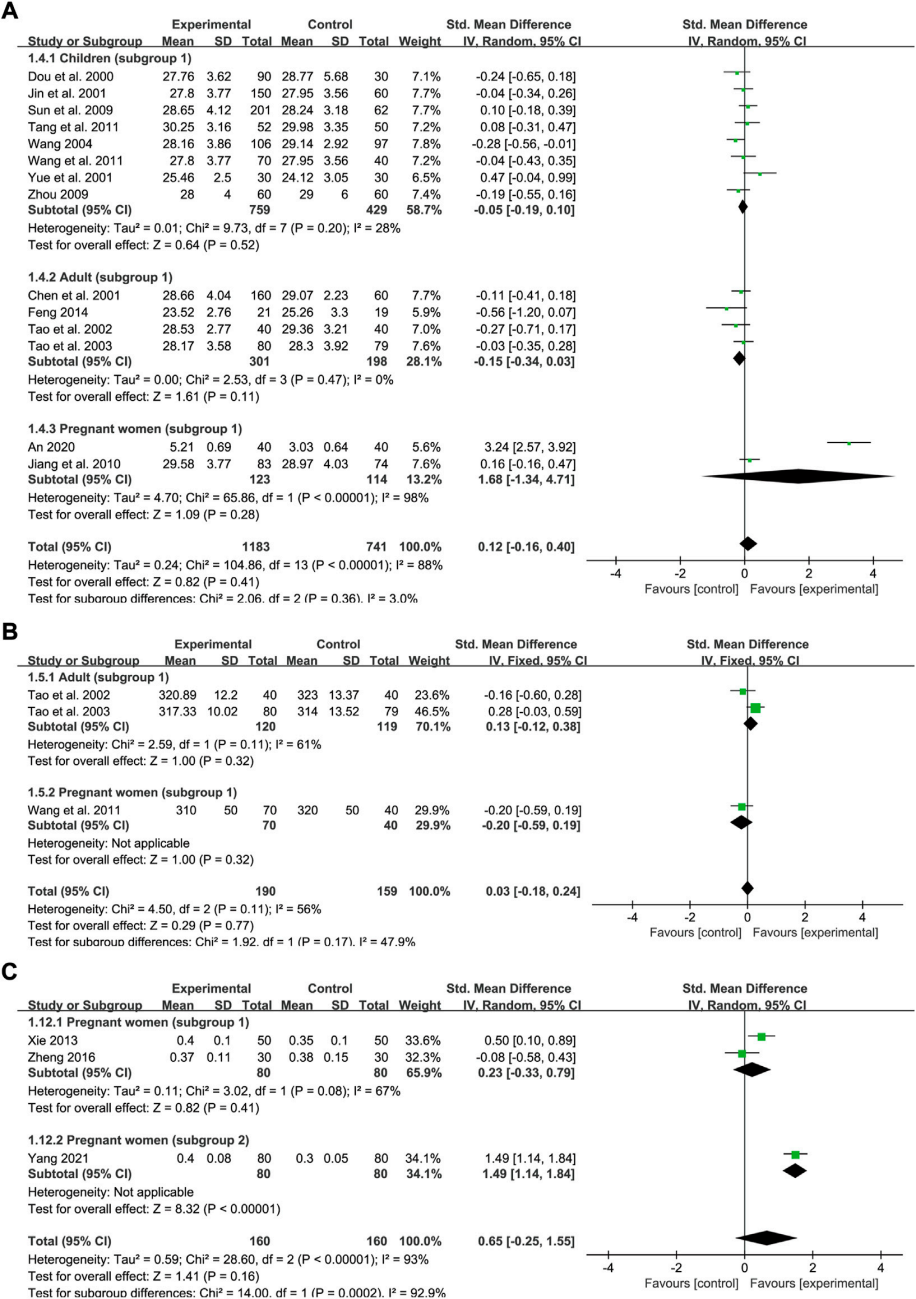
The forest plot assessing the effect of SXN on MCH **(A)**, MCHC **(B)**, and HCT **(C)**.

A total of 14 trials reported baseline and post-intervention data for MCH levels, of which eight trials included children (subgroup 1), four trials included adults (subgroup 1), and two trials included pregnant women (subgroup 1). The number of participants in the treatment group and control group was 1,183 and 741, respectively. The heterogeneity values were *p* < 0.00001, *I* ^2^ = 88% in total, *p* = 0.2, *I* ^2^ = 28% in children, *p* = 0.47, *I* ^2^ = 0% in adults and *p* < 0.00001, *I* ^2^ = 98% in pregnant women, thus a random-effects model was employed. Treatment courses across different groups were all 1 month approximately. Meta-regression analysis showed that the heterogeneity in different population groups was not significantly related to the number of cases (*p* = 0.672 in children; and *p* = 0.352 in adults), doses (*p* = 0.39 in children; and *p* = 0.352 in adults), and type of iron formulations (*p* = 0.98 in children). No significant difference was observed between the two groups regarding the effect on the MCH level in children [SMD = −0.05, 95% CI (−0.19, 0.1), *Z* = 0.64 (*p* = 0.52)], adults [SMD = −0.15, 95% CI (−0.34, 0.03), *Z* = 1.61 (*p* = 0.11)] as well as pregnant women [SMD = 1.68, 95% CI (−1.34, 4.71), *Z* = 1.09 (*p* = 0.28)]. The pooled effect size was SMD = 0.12, 95% CI (−0.16, 0.4), *Z* = 0.82 (*p* = 0.41) (Figure 6A). Egger’s test indicated that no publication bias was observed in children (*p* = 0.28) while publication bias might exist among the studies in adults (*p* = 0.039). Nevertheless, sensitivity analysis showed that the total effect sizes across all population groups were stable and the overall effect was not significantly influenced by any one study (Figures 9L,M).

### Mean corpuscular hemoglobin concentration

Only three studies compared the effect on MCHC levels between SXN treatment and iron supplementation (subgroup 1), of which two trials included adults and one trial included pregnant women. In all, 190 and 159 patients were included in the treatment and control groups, respectively. A random-effects model was used as the heterogeneity was high (*p* = 0.11, *I* ^2^ = 56% in total and *p* = 0.11, *I* ^2^ = 61% in adults). No marked difference in the MCHC was found between the two groups [SMD = 0.03, 95% CI (−0.18, 0.24), *Z* = 0.29 (*p* = 0.77) in total; SMD = 0.13, 95% CI (−0.12, 0.38), *Z* = 1 (*p* = 0.32) in adults; and SMD = −0.2, 95% CI (−0.59, 0.19), *Z* = 1 (*p* = 0.32) in pregnant women] (Figure 6B).

### Hematocrit

A total of three trials in pregnant women reported HCT data (two for subgroup 1 and one for subgroup 2) with 160 cases in the experimental group and 160 cases in the control group respectively. The heterogeneity values were *p* < 0.00001, *I* ^2^ = 93% in total and *p* = 0.23, *I* ^2^ = 67% in subgroup 1. Additionally, the results showed that, compared with the control treatment, SXN had a greater ameliorative effect on hematocrit [SMD = 0.65, 95% CI (−0.25, 1.55), *Z* = 1.41 (*p* = 0.16) overall; SMD = 0.23, 95% CI (−0.33, 0.79), *Z* = 0.82 (*p* = 0.41) for subgroup 1; and SMD = 1.49, 95% CI (1.14, 1.84), *Z* = 8.32 (*p* < 0.00001) for subgroup 2] (Figure 6C), although the difference was not significant. These results suggested that SXN and iron supplementation have comparable efficacies in raising HCT levels.

### Serum iron

**FIGURE 7:**
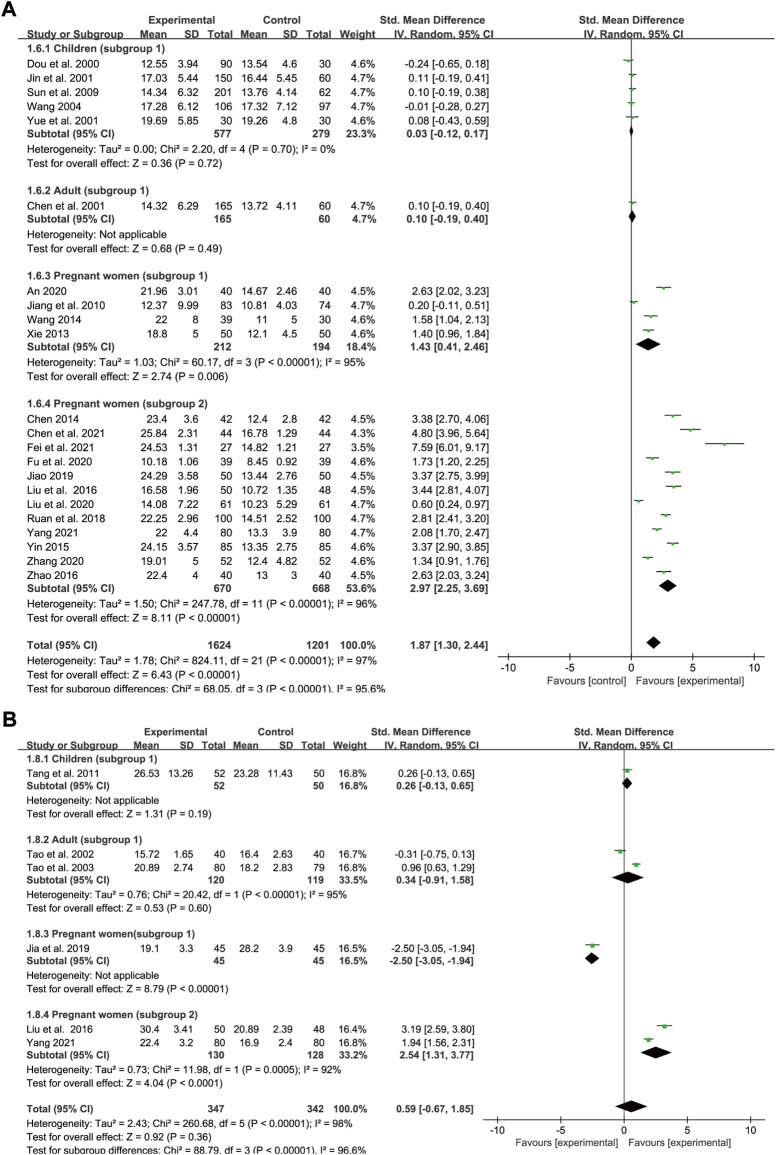
The forest plot assessing the effect of SXN on SI **(A)**, and SF **(B)**.

Data concerning SI status was extracted from 22 trials (five for subgroup 1 of children, one for subgroup 1 of adults, four for subgroup 1 of pregnant women and 12 for subgroup 2 of pregnant women). In all, 1,624 and 1,201 IDA patients were allocated to the experimental groups and iron supplement groups, respectively. Heterogeneity varied in the different groups (*p* < 0.00001, *I* ^2^ = 97% overall; *p* = 0.7, *I* ^2^ = 0% in children; *p* < 0.00001, *I* ^2^ = 95% in subgroup 1 of pregnant women; and *p* < 0.00001, *I* ^2^ = 96% in subgroup 2 of pregnant women). A random-effects model was applied for meta-analysis. All the interventions lasted for approximately 1 month. Meta-regression analysis showed that the heterogeneity in pregnant women was not significantly related to the number of cases (*p* = 0.14 in subgroup 1; and *p* = 0.268 in subgroup 2), doses (*p* = 0.479 in subgroup 2), and type of iron formulation (*p* = 0.216 in subgroup 2). The results showed that SXN had a greater ameliorative effect on SI levels [SMD = 1.87, 95% CI (1.3, 2.44), *Z* = 6.43 (*p* < 0.00001) in total; SMD = 0.03, 95% CI (−0.12, 0.17), *Z* = 0.36 (*p* = 0.72) in children; SMD = 0.1, 95% CI (−0.19, 0.4), *Z* = 0.68 (*p* = 0.49) in adults; SMD = 1.43, 95% CI (0.41, 2.46), *Z* = 2.74 (*p* = 0.006) in pregnant women (subgroup 1); and SMD = 2.97, 95% CI (2.25, 3.69), *Z* = 8.11 (*p* < 0.00001) in women (subgroup 2)] (Figure 7A). No publication bias was identified among the studies in children based on Egger’s test (*p* = 0.532), while publication bias was observed in different subgroups of pregnant women (Egger’s test: *p* = 0.03 and *p* = 0.004). Sensitivity analysis showed that the results were stable and no single study affected the total effect size across different population groups (Figures 9N–P).

### Serum ferritin

Data were extracted from six studies to evaluate the SF status, of which only one study evaluated SF levels in children, two studies in adults, one study in pregnant women (subgroup 1) and one study in pregnant women (subgroup 2). In all, 347 and 342 patients were enrolled in the treatment and control groups, respectively. The values for heterogeneity among the studies were *p* < 0.00001, *I* ^2^ = 98% in total; *p* < 0.00001, *I* ^2^ = 95% in adults; and *p* = 0.0005, *I* ^2^ = 92% in pregnant women (subgroup 2). The results showed that SXN had comparable effect on boosting SF levels compared with iron supplementation [SMD = 0.59, 95% CI (−0.67, 1.85), *Z* = 0.92 (*p* = 0.36) in total]. Based on the extracted data, no significant difference in SF levels was observed between the two groups in children [SMD = 0.26, 95% CI (−0.13, 0.65), *Z* = 1.31 (*p* = 0.19)] and adults [SMD = 0.34, 95% CI (−0.91, 1.58), *Z* = 0.53 (*p* = 0.6)]. SXN was less effective compared with iron supplementation in pregnant women [SMD = −2.5, 95% CI (−3.05, −1.94), *Z* = 8.79 (*p* < 0.00001)], while further analysis suggested that the effect of combined SXN/iron formulation treatment outperformed iron formulation monotherapy on this parameter in pregnant women [SMD = 2.54, 95% CI (1.31, 3.77), *Z* = 4.04 (*p* < 0.0001)] (Figure 7B).

### Total iron binding capacity

**FIGURE 8:**
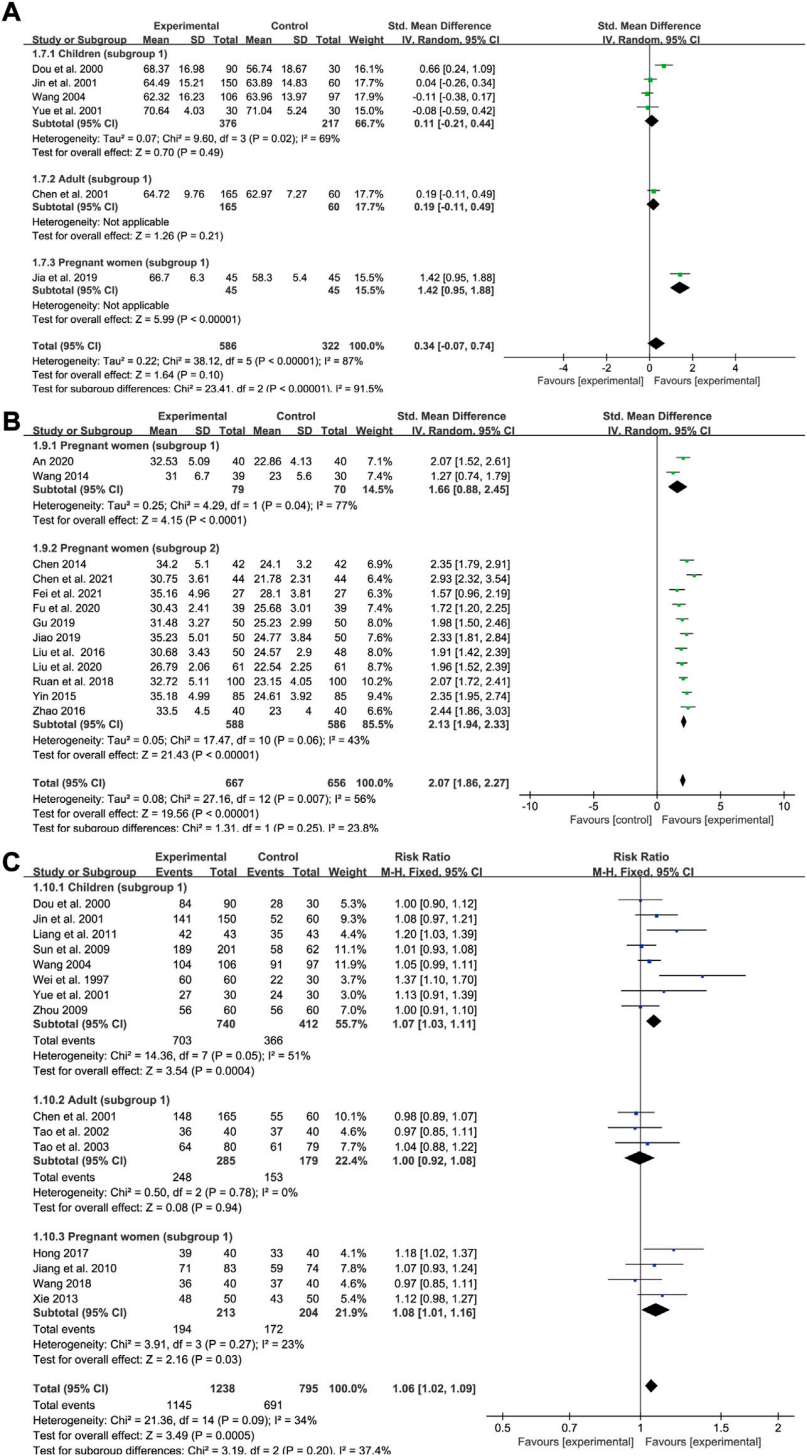
The forest plot assessing the effect of SXN on TIBC **(A)**, TSAT **(B)**, and total effective rate **(C)**.

**FIGURE 9:**
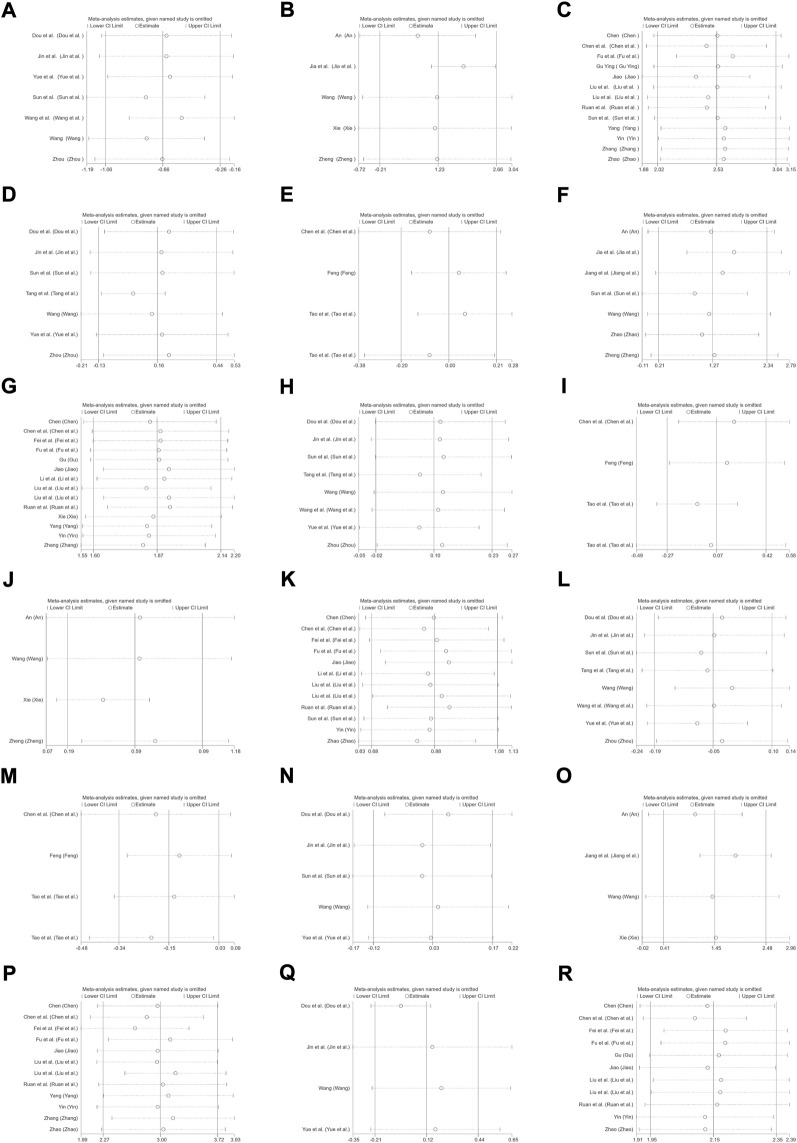
Sensitivity analysis plot. **(A)** Red blood cell count in children (subgroup 1); **(B)** Red blood cell count in pregnant women (subgroup 1); **(C)** Red blood cell count in pregnant women (subgroup 2); **(D)** Hb levels in children (subgroup 1); **(E)** Hb levels in adults (subgroup 1); **(F)** Hb levels in pregnant women (subgroup 1); **(G)** Hb levels in pregnant women (subgroup 2); **(H)** MCV levels in children (subgroup 1); **(I)** MCV levels in adults (subgroup 1); **(J)** MCV levels in pregnant women (subgroup 1); **(K)** MCV levels in pregnant women (subgroup 2); **(L)** MCH levels in children (subgroup 1); **(M)** MCH levels in adults (subgroup 1); **(N)** SI levels in children (subgroup 1); **(O)** SI levels in pregnant women (subgroup 1); **(P)** SI levels in pregnant women (subgroup 2); **(Q)** TIBC levels in children (subgroup 1); **(R)** TSAT levels in pregnant women (subgroup 2).

Data on TIBC levels was reported by six studies (four for subgroup 1 of children, one for subgroup 1 of adults and one for subgroup 1 of pregnant women). In all, 586 IDA patients were enrolled in the treatment group and 322 in the control group. A random-effects model was applied as heterogeneity was high across the studies (*p* < 0.00001, *I* ^2^ = 87% in total and *p* = 0.02, *I* ^2^ = 69% in children). The doses and courses of treatment were same across the studies in children and all of the participants in the control group applied Ferrous Succinate Tablets. Meta-regression analysis showed that the heterogeneity in children was not significantly related to the number of cases (*p* = 0.753). As shown in the forest plot graph in Figure 8A, the total effect size was SMD = 0.34, 95% CI (−0.07, 0.74), *Z* = 1.64 (*p* = 0.1). SXN had a fewer effect on TIBC levels in pregnant women compared with the iron formulation [SMD = 1.42, 95% CI (0.95, 1.88), *Z* = 5.99 (*p* < 0.00001)], while the efficacy in reducing TIBC levels was similar between the two groups among children [SMD = 0.11, 95% CI (−0.21, 0.44), *Z* = 0.7 (*p* = 0.49)]and adults [SMD = 0.19, 95% CI (−0.11, 0.49), *Z* = 1.26 (*p* = 0.21)]. Sensitivity analysis showed that the results were stable and no single study affected the total effect size in children (Figure 9Q), and no publication bias was found according to Egger’s test (*p* = 0.543).

### Transferrin saturation

Data for TSAT levels were extracted from 13 trials in pregnant women with IDA (two relating to subgroup 1 and 11 to subgroup 2). A total of 667 and 656 patients were included in the experimental and control groups, respectively. The heterogeneity results were as follows: *p* = 0.007, *I* ^2^ = 56% overall; *p* = 0.04, *I* ^2^ = 77% for subgroup 1; and *p* = 0.06, *I* ^2^ = 43% for subgroup 2. All the treatments lasted for approximately 1 month. Meta-regression analysis indicated that the heterogeneity in subgroup 2 was not significantly related to the number of cases (*p* = 0.778) or doses (*p* = 0.362), but was associated with the type of iron formulation (*p* = 0.001). Subgroup 2 was divided into five groups according to different types of iron formulations, nevertheless, the efficacy of SXN/iron formulation combination therapy in improving TSAT levels was similarly superior than that of iron supplementation monotherapies across different iron formulation groups (Supplementary Table S3). Overall, both SXN treatment alone and in combination with an iron supplement exhibited superior efficacy in increasing TSAT levels when compared with iron supplementation monotherapy, and the differences were statistically significant [SMD = 2.07, 95% CI (1.86, 2.27), *Z* = 19.56 (*p* < 0.00001) in total; SMD = 1.66, 95% CI (0.88, 2.45), *Z* = 4.15 (*p* < 0.0001) in subgroup 1; and SMD = 2.13, 95% CI (1.94, 2.33), *Z* = 21.43 (*p* < 0.00001) in subgroup 2] (Figure 8B). Egger’s test revealed that no publication bias existed among the studies in subgroup 2 (*p* = 0.646). Sensitivity analysis confirmed the stability of the results (Figure 9R).

### Total effective rate

We included 15 studies in the assessment of the total effective rate after SXN treatment, including eight trials in children (subgroup 1), three trials in adults (subgroup 1) and four trials in pregnant women (subgroup 1). A total of 1,238 and 795 patients were enrolled in the treatment and control groups, respectively. The heterogeneity among the studies was not notable (*p* = 0.09, *I* ^2^ = 34% in total; *p* = 0.05, *I* ^2^ = 51% in children; *p* = 0.78, *I* ^2^ = 0% in adults; and *p* = 0.27, *I* ^2^ = 23% in pregnant women), thus a fixed-effects model was applied. Egger’s test revealed that no publication bias existed among the studies in different population groups (*p* = 0.072 in children; *p* = 0.514 in adults; and *p* = 0.756 in pregnant women). The total effect size was RR = 1.06, 95% CI (1.02, 1.09), *Z* = 3.49 (*p* = 0.0005) (Figure 8C). No significant difference in the total effective rate was observed between SXN treatment and iron supplementation in adults [RR = 1, 95% CI (0.92, 1.08), *Z* = 0.08 (*p* = 0.94)], while SXN exhibited a greater capacity for increasing the total effective rate compared with the control group in children [RR = 1.07, 95% CI (1.03, 1.11), *Z* = 3.54 (*p* = 0.0004)] and pregnant women [RR = 1.08, 95% CI (1.01, 1.16), *Z* = 2.16 (*p* = 0.03)], implying that SXN therapy elicited superior responses in the remission of clinical symptoms and improvement of clinical parameters among children and pregnant women with IDA.

### Adverse event rate

**FIGURE 10:**
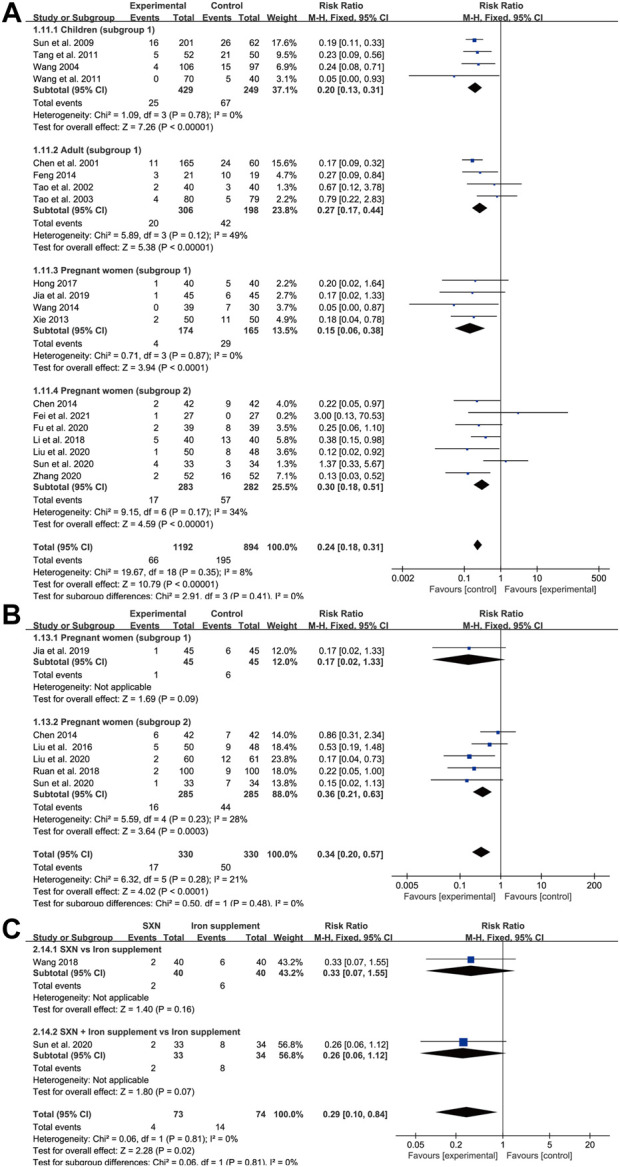
The forest plot for adverse event rates **(A)**, adverse pregnancy outcomes **(B)** and anemia recurrence rates after the treatment till delivery **(C)** in pregnant women with IDA.

Among the 39 trials, 16 did not report complete results for the occurrence of adverse events, while four trials reported the absence of adverse events in both groups. The remaining 19 trials reported a total of 261 cases (41 in the treatment group and 128 in the control group) of rash or gastrointestinal reactions, including nausea, vomiting, abdominal pain, diarrhea, and constipation (Figure 10A). Overall, there were 66 instances of adverse events in 1,192 patients in the treatment group and 195 in 894 patients from the control group. A fixed-effects model was applied as heterogeneity was low across the studies (*p* = 0.35, *I* ^2^ = 8% in total; *p* = 0.78, *I* ^2^ = 0% in children; *p* = 0.12, *I* ^2^ = 49% in adults; *p* = 0.87, *I* ^2^ = 0% in subgroup 1 of pregnant women; and *p* = 0.17, *I* ^2^ = 34% in subgroup 2 of pregnant women). Meta-analysis results indicated that the adverse event rate in the treatment group was markedly lower than that of the control group, highlighting that SXN and SXN/iron supplementation combination treatment was superior at reducing the risk of adverse reactions compared with iron supplementation monotherapy [RR = 0.24, 95% CI (0.18, 0.31), *Z* = 10.79 (*p* < 0.00001) in total; RR = 0.2, 95% CI (0.13, 0.31), *Z* = 7.26 (*p* < 0.00001) in children; RR = 0.27, 95% CI (0.17, 0.44), *Z* = 5.38 (*p* < 0.00001) in adults; RR = 0.15, 95% CI (0.06, 0.38), *Z* = 3.94 (*p* < 0.0001) in pregnant women (subgroup 1); and RR = 0.3, 95% CI (0.18, 0.51), *Z* = 4.59 (*p* < 0.00001) in pregnant women (subgroup 2)]. No publication bias was observed among the studies based on Egger’s test [*p* = 0.51 in children; *p* = 0.1 in adults; *p* = 0.259 in pregnant women (subgroup 1); and *p* = 0.818 in pregnant women (subgroup 2)].

### Adverse pregnancy outcomes

A total of six trials in pregnant women (one relating to subgroup 1 and five to subgroup 2) reported adverse pregnancy outcomes among 660 pregnant women with IDA (330 in the treatment group and 330 in the control group). Reported adverse pregnancy outcomes consisted of premature delivery, postpartum hemorrhage, hypertension in pregnancy, fetal distress, and low birth weight. A fixed-effects model was adopted due to high homogeneity across the studies (*p* = 0.28, I^2^ = 21% in total, *p* = 0.23, I^2^ = 28% in subgroup 2). Egger’s test indicated that publication bias might exist among the subgroup 2 (*p* = 0.04). As shown in Figure 10B, compared with the control group, SXN treatment and combined therapy with SXN could effectively reduce the risk of adverse pregnancy incidences [RR = 0.34, 95% CI (0.2, 0.57), *Z* = 4.02 (*p* < 0.0001) in total, RR = 0.17, 95% CI (0.02, 1.33), *Z* = 1.69 (*p* = 0.09) in subgroup 1, RR = 0.36, 95% CI (0.21, 0.63), *Z* = 3.64 (*p* = 0.0003) in subgroup 2], rendering SXN treatment a safer regimen than iron supplementation for patients with IDA who are also pregnant.

### Anemia recurrence rate

There were two trials (one of subgroup 1, one of subgroup 2) in IDA patients with pregnancy that reported anemia recurrence rates during pregnancy after the treatment till delivery. There was no heterogeneity between two studies (*p* = 0.81, I^2^ = 0%). Meta-analysis results showed RR = 0.29, 95% CI (0.1, 0.84), Z = 2.28 (*p* = 0.02) in total; RR = 0.33, 95% CI (0.07, 1.55)], Z = 1.4 (*p* = 0.16) in subgroup 1; and RR = 0.26, 95% CI (0.06, 1.12)], Z = 1.8 (*p* = 0.07) in subgroup 2 (Figure 10C), providing convincing evidence for SXN-contained therapy with respect of steady efficiency and high safety.

## Meta-analysis results relating to the preventive effect of Shengxuening tablet on iron deficiency anemia during pregnancy

### Red blood cell count

**FIGURE 11:**
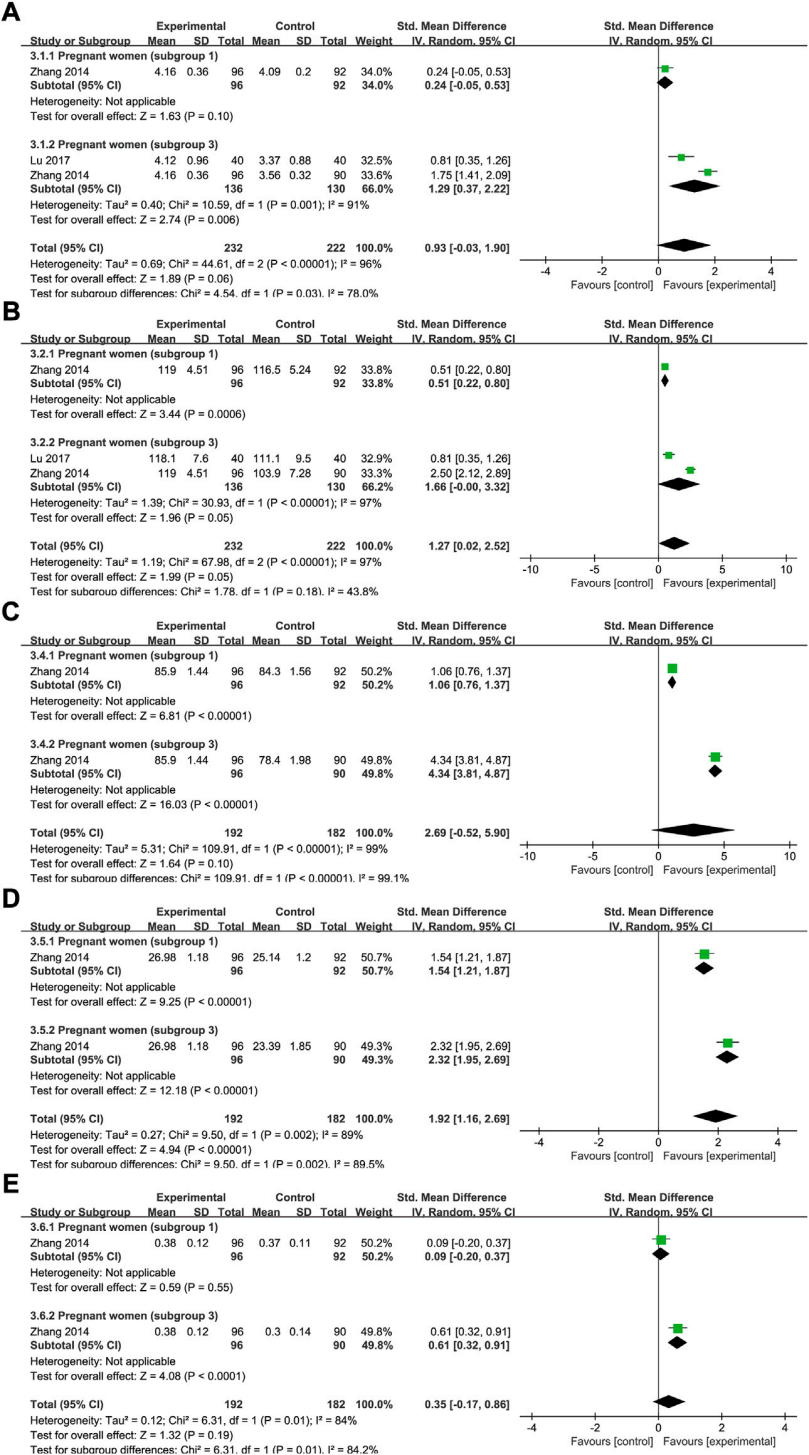
The forest plot assessing the effect of SXN in preventing IDA during pregnancy (involving subgroup 1 and subgroup 3) in terms of RBC count **(A)**, Hb **(B),** MCV **(C)** MCH **(D)**, and HCT **(E)**.

There were three trials that included data for RBC counts under SXN intervention for 232 cases of IDA and 222 controls. The results of the heterogeneity analysis were *p* < 0.00001, *I* ^2^ = 96% in total and *p* = 0.001, *I* ^2^ = 91% in subgroup 3. The total effect size was SMD = 0.93, 95% CI (−0.03, 1.9), *Z* = 1.89 (*p* = 0.06). SXN effectively prevented RBC count dropping in subgroup 3 [SMD = 1.29, 95% CI (0.37, 2.22), *Z* = 2.74 (*p* = 0.006), and exerted comparable preventive effect on RBC count to iron supplementations in subgroup 1 [SMD = 0.24, 95% CI (−0.05, 0.53), *Z* = 1.63 (*p* = 0.1) (Figure 11A), implying that the application of SXN during pregnancy might be a promising strategy to avoid RBC count dropping.

### Hemoglobin

Data on Hb levels were reported by three trials (232 patients in the treatment group and 222 in the control group). The heterogeneity results were as follows: *p* < 0.00001, I^2^ = 97% in total; and *p* < 0.00001, I^2^ = 97% in subgroup 3. The results of the meta-analysis demonstrated that, compared with the controls, SXN treatment improved the Hb level [SMD = 1.27, 95% CI (0.02, 2.52), *Z* = 1.99, (*p* = 0.05) in total; SMD = 0.51, 95% CI (0.22, 0.8), *Z* = 3.44, (*p* = 0.0006) in subgroup 1; and SMD = 1.66, 95% CI (0, 3.32), *Z* = 1.96, (*p* = 0.05) in subgroup 3] (Figure 11B).

### Other iron deficiency anemia-related clinical parameters

Data for MCV, MCH, HCT, and adverse events were extracted from two clinical trials (one relating to subgroup 1 and one to subgroup 3) in the same study. The heterogeneity results were as follows: *p* < 0.00001, I^2^ = 99% for MCV; *p* = 0.002, I^2^ = 89% for MCH; and *p* = 0.01, I^2^ = 84% for HCT. As shown in Figures 11C–E, compared with the blank control, SXN treatment exhibited superior efficacy in raising MCV [SMD = 4.34, 95% CI (3.81, 4.87), *Z* = 16.03, (*p* < 0.00001)], MCH [SMD = 2.32, 95% CI (1.95, 2.69), *Z* = 12.18, (*p* < 0.00001)] and HCT [SMD = 0.61, 95% CI (0.32, 0.91), *Z* = 1.32, (*p* < 0.0001)]. SXN treatment also outperformed iron formulations on MCV [SMD = 1.06, 95% CI (0.76, 1.37), *Z* = 6.81, (*p* < 0.00001)] and MCH levels [SMD = 1.54, 95% CI (1.21, 1.87), *Z* = 9.25, (*p* < 0.00001)]. Compared to the iron supplementation, SXN treatment also exerted a strong ameliorative effect on HCT levels [SMD = 0.09, 95% CI (−0.2, 0.37), *Z* = 0.59, (*p* = 0.55)] and adverse events [RR = 0.47, 95% CI (0.2, 1.1)], Z = 1.75(*p* = 0.08), however, these effects were not statistically significant.

### The incidence of iron deficiency anemia

**FIGURE 12:**
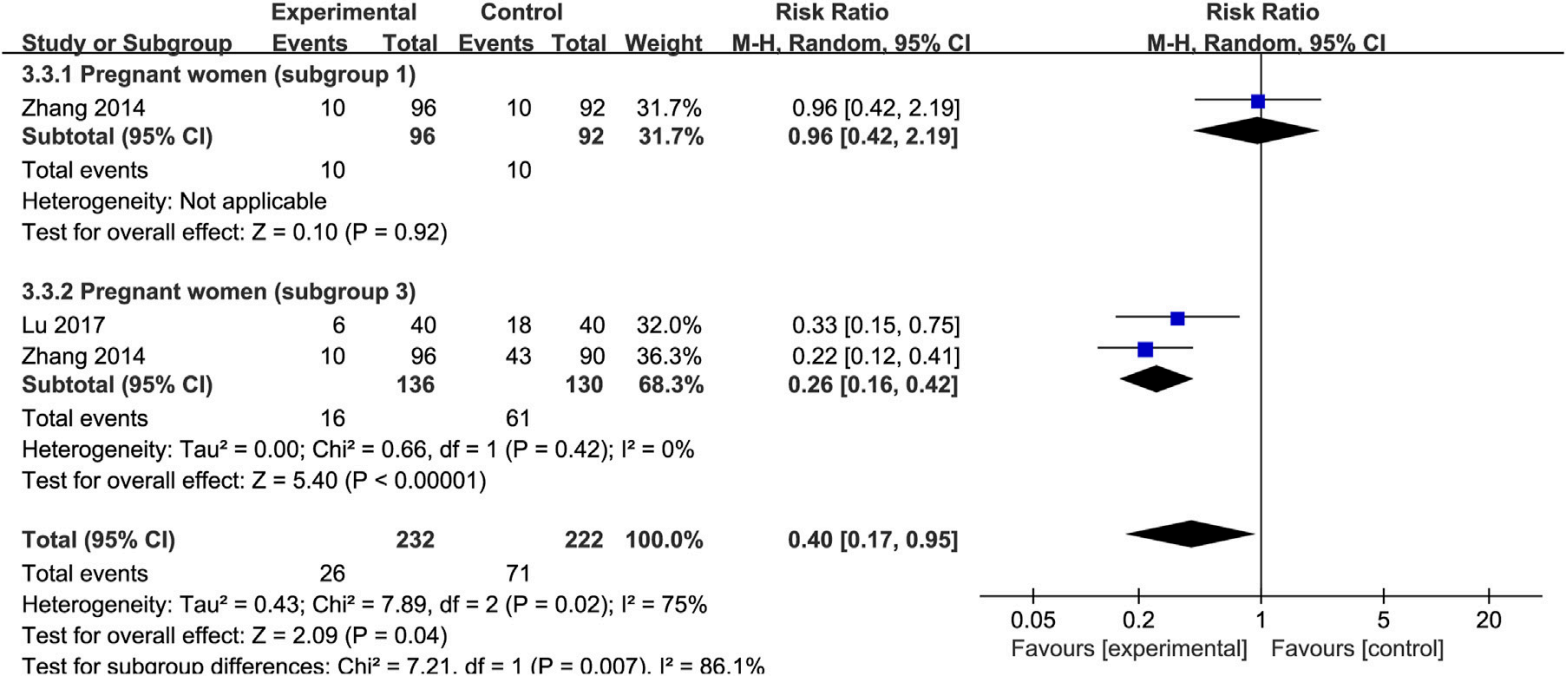
The forest plot assessing the efficiency of SXN in reducing the risk of IDA incidence during pregnancy.

The effect of SXN on the risk of IDA incidence during pregnancy was evaluated in three trials involving 232 patients with IDA and 222 controls. The results of the heterogeneity analysis were *p* = 0.04, I^2^ = 75% in total, *p* = 0.42, I^2^ = 0% in subgroup 3. As Figure 12 shown, the total effect size was RR = 0.4, 95% CI (0.17, 0.95], Z = 2.09 (*p* = 0.04). SXN greatly reduced the risk for IDA compared to the blank control group [RR = 0.26, 95% CI (0.16, 0.42)], Z = 5.4 (*p* < 0.00001)], while the preventive ability of SXN treatment was comparable to that of iron formulation [RR = 0.96, 95% CI (0.42, 2.19)], Z = 0.1 (*p* = 0.92)]. The results revealed the high efficiency of SXN in reducing IDA risk, and that SXN therapy has the potential to be a reliable intervention for the prevention of IDA during pregnancy.

## Discussion

IDA is a significant public health problem, with approximately 1.24 billion people affected by the disease worldwide (Vanobberghen et al., 2021). SXN is a Chinese patent medicine extracted from silkworm excrement. Silkworm excrement is a long-history traditional Chinese medicine, which was recorded in many Chinese ancient medical books, including the Compendium of Materia Medica and Supplement to Medica (Wei et al., 1997; Wei et al., 2005). It possesses sweet and pungent taste and is warm in nature, has effects of removing blood stasis, replenishing qi and nourishing blood and has been widely documented to treat anemia with deficiency of both qi and blood (Chen, 2014; Ding et al., 2019). Therefore, SXN is suitable for anemia with qi and blood deficiency, it has been widely used for the treatment of hematopoietic diseases, including renal anemia, aplastic anemia, IDA, and idiopathic thrombocytopenic purpura (Shao et al., 2007). Several studies have reported the therapeutic effect of SXN on IDA, which may involve two main mechanisms.

First, SXN can promote hematopoiesis via different signaling pathways. Studies have demonstrated that SXN increased the synthesis of erythropoietin in rats, thereby ameliorating renal anemia in the animals (Mei et al., 2021). Furthermore, SXN promoted the restoration of hemopoietic function in models of myelosuppression by enhancing the secretion of hematopoietic factors and activating the JAK2/STAT3 pathway (Ding et al., 2021). Moreover, another study reported that SXN could also significantly upregulate the expression of granulocyte macrophage colony stimulating factor and stem cell factor in bone marrow cells, thereby alleviating cyclophosphamide-mediated myelosuppression (Huang et al., 2016).

SXN can also facilitate iron absorption by regulating iron metabolism. One study found that silkworm excrement extract can inhibit hepcidin expression by blocking the BMP6/SMAD4 and IL-6/STAT3 pathways in a rat model of renal anemia (Mei et al., 2021). Similarly, Liu and Li (2020) reported that a SXN-containing regimen exerted therapeutic effects on IDA by inhibiting hepcidin expression and regulating iron metabolism. In addition, fingerprint analysis revealed that the main ingredient in SXN is a ferrous derivative mainly composed of Fe chlorin p6, Fe chlorin e6, and Fe isochlorin e4 (Nie et al., 2013). Recent pharmacology-based studies have reported that chlorophyll derivatives and sodium iron chlorophyllin are the main components of SXN. Both are natural porphyrins that can be directly absorbed by intestinal mucosal cells with high efficiency and good bioavailability, and induce only mild gastrointestinal irritation; consequently, they are rarely associated with adverse reactions (Ruan et al., 2018; Fei et al., 2021).

### Main findings

A total of 39 randomized controlled clinical trials comprising 4,562 cases were included in our analysis. Ten of these studies were performed in children <four years of age and 29 involved adults, 22 of which were conducted on pregnant women.

Although the assessment of the effect of SXN on IDA-related parameters were different to a small extent across various population groups, the overall performance of SXN in preventing and treating IDA was satisfying and convincing. The total efficiency of SXN was superior than the control group in improving RBC count, Hb, MCV, SI, and TSAT levels. Besides, the total effects of SXN to improve MCH, MCHC, HCT, SF levels and reduce the TIBC was comparable to that of iron supplementation. Moreover, SXN significantly raised the total effective rates of IDA compared to oral iron formulations. Oral iron treatment is the first-line treatment for IDA; however, the adherence of patients to iron treatment can be affected by a high frequency of gastrointestinal-related side-effects (Lewkowitz and Tuuli, 2019). Our data demonstrated that SXN intervention attained a similarly satisfactory clinical response as iron preparations, but was associated with fewer adverse events. Furthermore, the therapeutic efficacy of SXN treatment combined with iron supplementation was better than that of iron supplementation alone and was linked to a markedly reduced risk of side-effects. These findings indicated that SXN can be recommended as an ideal strategy for decreasing the side-effect burden of iron treatment in IDA without negatively affecting outcomes.

According to the World Health Organization (WHO), more than one-third of pregnant women suffer from IDA worldwide and post-partum anemia is associated with adverse maternal and neonatal outcomes (Vanobberghen et al., 2021). Our analysis showed that, compared with iron supplementation, SXN treatment and combined therapy with SXN contributed to a lower incidence of adverse pregnancy events and anemia recurrence rates in pregnant women with IDA. Moreover, regarding prevention, SXN administration also exhibited superior efficacy in raising RBC counts, Hb, MCV, and HCT levels and reducing the number of adverse events; however, these effects were not statistically significant. SXN also performed better in decreasing the risk of IDA incidence during pregnancy, providing convincing evidence that SXN may represent an optimum regimen for IDA patients, especially for the poor-tolarated populations who are at risk of IDA or have been diagnosed with IDA.

### Comparisons with previous meta-analyses

To date, only one meta-analysis has evaluated the therapeutic efficacy of SXN in the treatment of IDA during pregnancy (Chen et al., 2018). That study included 11 randomized controlled clinical trials between 2008 and 2018 involving 1,617 patients and the estimated outcome only involved the overall effective rate. The results may have been influenced by the limited number of references and lack of methodology, such as analysis of publication bias and sensitivity. Besides, the study did not evaluate IDA-related clinical parameters, which can provide more precise information for the appraisal of clinical efficacy, nor did it account for adverse events. Thus, the safety of SXN was not systematically assessed.

In contrast, we enrolled patients with IDA from a wide range of populations (children, adults, and pregnant women), which allowed for a more comprehensive estimation of the efficacy of SXN. We also included recently published studies and examined the stability and heterogeneity among studies employing the appropriate statistical methods. Moreover, we investigated several essential clinical indicators to build convincing evidence for the final conclusions, and also evaluated several adverse outcomes such as the numbers of adverse events, adverse pregnancy outcomes, and recurrence rates, which enabled us to assess the safety of the medications.

### Limitations of the study

This meta-analysis has several limitations. First, only 16 of the trials reported the method used for random sequence generation and only one study reported the method employed for blinding. Furthermore, none of the studies described the specific methods used for allocation concealment. Consequently, the methodological quality of the included studies was not satisfactory. Besides, although we performed subgroup analysis and meta regression analysis to identify the source of heterogeneity, high heterogeneity could not be avoided, which may have influenced the pooling results. In addition, the number of references in some subgroups was limited, which may have negatively affected the results.

## Conclusion

Based on the meta-analysis results from 39 trials, although the effect size of SXN for IDA-related parameters were different across population groups, the total efficiency of SXN and combined SXN/iron formulation intervention for the prevention and treatment of IDA was convincing, evidenced by comparable or even superior effect of SXN in blood routine index and iron status compared to iron formulations. SXN was also safer and had a lower adverse events burden compared to the routine oral iron supplementations, suggesting that SXN was a reliable treatment option for IDA. Further research is expected to overcome the limitation of this study and provide more robust evidence regarding the efficiency and safety of SXN.

## Data Availability

The raw data supporting the conclusion of this article will be made available by the authors, without undue reservation.
