# Supplementary material for: The efficiency and safety of Shengxuening tablet on treating and preventing iron deficiency anemia: A systematic review and meta-analysis
Source: Front Pharmacol. 2022 Nov 3;13:1029641. doi: 10.3389/fphar.2022.1029641 (PMC9669986; doi:10.3389/fphar.2022.1029641)
Supplement: Supplementary file 1 [file DataSheet1.docx]

Supplementary Material

# Supplementary Table 1. The characteristics of the included studies

| **Studies** | **Populations** | **No.(T/C)** | **Treatment group** | **Control group** | **Duration** | **Dose** | **Subgroup** | **Outcomes** |
| --- | --- | --- | --- | --- | --- | --- | --- | --- |
| An Hong (2020) | Pregnant women | 40/40 | SXN | Ferrous Succinate Tablet | 1 month | 0.5g tid | 1 | ①②③⑦⑩ |
| Chen Fang (2014) | Pregnant women | 42/42 | SXN + Compound Ferrous Sulfate and Folic Acid Tablet | Compound Ferrous Sulfate and Folic Acid Tablet | 1 month | 0.5g tid | 2 | ①②③⑦⑩⑫⑬ |
| Chen Xiaozhe et al. (2021) | Pregnant women | 44/44 | SXN + Ferrous Succinate Tablet | Ferrous Succinate Tablet | 4 weeks | 0.5g tid | 2 | ①②③⑦⑩ |
| Chen Yunliang et al. (2001) | Adult | 165/60 | SXN | Ferrous Succinate Tablet | 4 weeks | 0.5g tid | 1 | ①②③④⑦⑨⑪⑫ |
| Dou Jianwei et al. (2000) | Children | 90/30 | SXN | Ferrous Succinate Tablet | 30 days | 0.25g tid | 1 | ①②③④⑦⑨⑪ |
| Fei Lixiao et al. (2021) | Pregnant women | 27/27 | SXN + Ferrous Lactate Oral Solution | Ferrous Lactate Oral Solution | 4 weeks | 0.5g bid | 2 | ②③⑦⑩⑫ |
| Fu Yanhong et al. (2020) | Pregnant women | 39/39 | SXN + Iron Dextran Dispersible Tablet | Iron Dextran Dispersible Tablet | 30 days | 0.5g tid | 2 | ①②③⑦⑩⑫⑬ |
| Gu Ying (2019) | Pregnant women | 50/50 | SXN + Ferrous Succinate Tablet | Ferrous Succinate Tablet | 4 weeks | 0.5g bid | 2 | ①②⑩⑫ |
| Hong Hua (2017) | Pregnant women | 40/40 | SXN | Ferrous Sulfate Tablet | 1 month | 0.5g tid | 1 | ⑪⑫ |
| Jia Aiying and Wang (2019) | Pregnant women | 45/45 | SXN | Ferrous Succinate Tablet | 3 months | 0.5g bid /0.5g tid | 1 | ①②⑧⑨⑫ |
| Jiang Dan et al. (2010) | Pregnant women | 84/84 | SXN | Compound Ferrous Sufate Granules | 4 weeks | 0.5g tid | 1 | ②④⑦⑪ |
| Jiao Zhihong (2019) | Pregnant women | 50/50 | SXN + Ferrous Succinate Tablet | Ferrous Succinate Tablet | 28 days | 0.5g tid | 2 | ①②③⑦⑩ |
| Jin Jianzhong et al. (2001) | Children | 150/60 | SXN | Ferrous Succinate Tablet | 4 weeks | 0.25g tid | 1 | ①②③④⑦⑨⑪ |
| Yue Qin et al. (2001) | Children | 30/30 | SXN | Ferrous Succinate Tablet | 30 days | 0.25g tid | 1 | ①②③④⑦⑨⑪ |
| Li Zhihui and Wang (2018) | Pregnant women | 40/40 | SXN + Ferrous Succinate Tablet | Ferrous Succinate Tablet | 1 month | 0.5g tid | 2 | ②③⑫ |
| Liang Ping and Lou (2011) | Children | 43/43 | SXN | Ferrous Gluconate | 4 weeks | 0.025g tid | 1 | ⑪ |
| Liu Liheng et al. (2016) | Pregnant women | 50/50 | SXN + Polysaccharide Iron Complex Capsule | Polysaccharide Iron Complex Capsule | 4 weeks | 0.5g bid /0.5g tid | 2 | ①②③⑦⑧⑩⑫⑬ |
| Liu Menghui and Li (2020) | Pregnant women | 61/61 | SXN + Polysaccharide Iron Complex Capsule | Polysaccharide Iron Complex Capsule | 4 weeks | 0.5g bid | 2 | ①②③⑦⑩⑬ |
| Ruan Shengming et al. (2018) | Pregnant women | 100/100 | SXN + Ferrous succinate | Ferrous Succinate Tablet | 28 days | 0.5g tid | 2 | ①②③⑦⑩⑬ |
| Sun Liying and Wang (2020) | Pregnant women | 33/34 | SXN + Iron Proteinsuccinylate Oral Solution | Iron Proteinsuccinylate Oral Solution | 4 weeks | 0.5g tid | 2 | ①②③⑦⑩⑫⑬⑭ |
| Sun Xiaojing et al. (2009) | Children | 201/62 | SXN | Ferrous Sulfate Tablet | 4 weeks | 0.125g tid | 1 | ①②③④⑦⑪⑫ |
| Tang Limin et al. (2011) | Children | 52/50 | SXN | Ferrous Sulfate Tablet | 30 days | 0.125g tid /0.25g tid | 1 | ②③④⑧⑫ |
| Tao Hong et al. (2002) | Adult | 40/40 | SXN | Ferrous Sulfate and Vitamin Complex Sustained-release Tablet | 4 weeks | 0.5g tid | 1 | ①②③④⑤⑧⑪⑫ |
| Tao Hong et al. (2003) | Adult | 80/79 | SXN | Ferrous Sulfate and Vitamin Complex Sustained-release Tablet | 4 weeks | 0.5g tid | 1 | ②③④⑤⑧⑪⑫ |
| Wang Hong (2018) | Pregnant women | 40/40 | SXN | Ferrous Succinate Tablet | 2 months | 0.5g bid/0.5g tid | 1 | ⑪⑭ |
| Wang Jingmin and Feng (2011) | Children | 70/40 | SXN | Ferrous Sufate Granules | 4 weeks | 0.25g tid | 1 | ①③④⑤⑫ |
| Wang Lianfang (2004) | Children | 106/97 | SXN | Ferrous Succinate Tablet | 4 weeks | 0.25g tid | 1 | ①②③④⑦⑨⑪⑫ |
| Wang Yajun (2014) | Pregnant women | 39/30 | SXN | Polysaccharide Iron Complex Capsule | 4 weeks | 0.5g tid | 1 | ①②③⑦⑩⑫ |
| Xie Zhuo (2013) | Pregnant women | 50/50 | SXN | Ferrous Sulfate Tablet | 4 weeks | 0.5g tid | 1 | ①②③⑥⑦⑪⑫ |
| Yang Li (2021) | Pregnant women | 80/80 | SXN + Ferrous Succinate Tablet | Ferrous Succinate Tablet | 4 weeks | 0.5g bid | 2 | ①②⑥⑦⑧⑫ |
| Yin Qinglan (2015) | Pregnant women | 85/85 | SXN + Ferrous Succinate Tablet | Ferrous Succinate Tablet | 4 weeks | 0.5g tid | 2 | ①②③⑦⑩⑫ |
| Zhang Jianhua (2020) | Pregnant women | 52/52 | SXN + Ferrous Succinate Tablet | Ferrous Succinate Tablet | 4 weeks | 0.25g tid | 2 | ①②⑦⑫ |
| Zhao Xia (2016) | Pregnant women | 40/40 | SXN + Compound Ferrous Sulfate and Folic Acid Tablet | Compound Ferrous Sulfate and Folic Acid Tablet | 1 month | 0.5g bid | 2 | ①②③⑦⑩ |
| Zheng Hong (2016) | Pregnant women | 30/30 | SXN | Polysaccharide Iron Complex Capsule | From 28th gestational week till delivery | 0.5g bid | 1 | ①②③⑥ |
| Zhou Yunlan (2009) | Children | 60/60 | SXN | Ferrous Sulfate Tablet | 4 weeks | 0.25g tid | 1 | ①②③④⑪ |
| Feng Lei (2014) | Adult | 21/19 | SXN | Ferrous Sulfate Tablet | 1 month | 0.5g tid | 1 | ②③④⑫ |
| Wei Kemin et al. (1997) | Children | 60/30 | SXN | Ferrous Sulfate Tablet | 30 days | 0.25g tid | 1 | ⑪⑫ |
| Zhang Ruijie (2014) | Pregnant women | 100/100 | SXN | Ferrous Succinate Tablet | 12 weeks | 0.5g bid | 1 | ②⑧⑮ |
| Zhang Ruijie (2014) | Pregnant women | 100/100 | SXN |  | 12 weeks | 0.5g bid | 3 | ②⑧⑮ |
| Lu Xia (2017) | Pregnant women | 40/40 | SXN |  | From 16th gestational week till delivery | 0.5g bid /0.5g tid | 3 | ①②⑦⑧⑮ |
| Wang Lei (2016) | Pregnant women | 68/68 | SXN |  | 8 weeks | 0.5g bid | 3 | ①②③④⑥⑮ |

Note：No., number of participants; T, treatment group; C, control group; SXN, Shengxuening Tablet; Outcome indicators: ①RBC count; ②Hb; ③MCV; ④MCH; ⑤MCHC; ⑥HCT; ⑦SI; ⑧SF; ⑨TIBC; ⑩TSAT; ⑪IDA effective rate; ⑫Adverse events; ⑬Adverse pregnancy outcomes; ⑭Anemia recurrence rate; ⑮Incidence of IDA

# Supplementary Table 2. Assessment methods for the various outcomes

| **Included studies** | **Blood samples** | | **Routine blood test** | **SI** | **TSAT** |
| --- | --- | --- | --- | --- | --- |
| An Hong (2020) | venous blood | blood cell analyzer | | automatic biochemistry analyzer | ferritin immunoelectron microscopy |
| Chen Fang (2014) | venous blood |  | |  |  |
| Chen Xiaozhe et al. (2021) |  | blood cell analyzer | | automatic biochemistry analyzer | ferritin immunoelectron microscopy |
| Chen Yunliang et al. (2001) |  |  | |  |  |
| Dou Jianwei et al. (2000) |  |  | |  |  |
| Fei Lixiao et al. (2021) | venous blood | blood cell analyzer | | automatic biochemistry analyzer | ferritin immunoelectron microscopy |
| Fu Yanhong et al. (2020) | venous blood |  | |  |  |
| Gu Ying (2019) |  |  | |  |  |
| Hong Hua (2017) |  |  | |  |  |
| Jia Aiying and Wang (2019) |  |  | |  |  |
| Jiang Dan et al. (2010) |  |  | |  |  |
| Jiao Zhihong (2019) |  |  | |  |  |
| Jin Jianzhong et al. (2001) |  |  | |  |  |
| Yue Qin et al. (2001) |  |  | |  |  |
| Li Zhihui and Wang (2018) | venous blood | blood cell analyzer | |  |  |
| Liang Ping and Lou (2011) |  |  | |  |  |
| Liu Liheng et al. (2016) |  | blood cell analyzer | |  |  |
| Liu Menghui and Li (2020) | venous blood | blood cell analyzer | | automatic biochemistry analyzer | ferritin immunoelectron microscopy |
| Ruan Shengming et al. (2018) | venous blood | blood cell analyzer | | automatic biochemistry analyzer | ferritin immunoelectron microscopy |
| Sun Liying and Wang (2020) | venous blood | blood cell analyzer | |  |  |
| Sun Xiaojing et al. (2009) |  |  | |  |  |
| Tang Limin et al. (2011) |  |  | |  |  |
| Tao Hong et al. (2002) |  |  | |  |  |
| Tao Hong et al. (2003) |  |  | |  |  |
| Wang Hong (2018) |  |  | |  |  |
| Wang Jingmin and Feng (2011) |  | blood cell analyzer | |  |  |
| Wang Lianfang (2004) |  |  | |  |  |
| Wang Yajun (2014) |  |  | |  |  |
| Xie Zhuo (2013) |  |  | |  |  |
| Yang Li (2021) |  |  | |  |  |
| Yin Qinglan (2015) |  | blood cell analyzer | | automatic biochemistry analyzer |  |
| Zhang Jianhua (2020) |  |  | |  |  |
| Zhao Xia (2016) |  |  | |  |  |
| Zheng Hong (2016) |  |  | |  |  |
| Zhou Yunlan (2009) | venous blood |  | |  |  |
| Feng Lei (2014) |  |  | |  |  |
| Wei Kemin et al. (1997) |  |  | |  |  |
| Zhang Ruijie (2014) |  |  | |  |  |
| Zhang Ruijie (2014) |  |  | |  |  |
| Lu Xia (2017) |  | blood cell analyzer | | automatic immune analyzer |  |
| Wang Lei (2016) |  |  | |  |  |

# Supplementary Table 3. The main compositions of the 10 oral iron formulations

| Iron formulations | Main composition |
| --- | --- |
| Ferrous Succinate Tablets | Ferrous Succinate |
| Ferrous Sulfate Tablets | Ferrous Sulfate |
| Compound Ferrous Sulfate and Folic Acid Tablets | Ferrous Sulfate; Folic Acid |
| Ferrous Lactate Oral Solution | Ferrous Lactate; Vitamin C |
| Iron Dextran Dispersible Tablets | Iron dextran |
| Compound Ferrous Sufate Granules | Ferrous Sulfate |
| Ferrous Gluconate Tablets | Ferrous Gluconate |
| Polysaccharide Iron Complex Capsule | Polysaccharide iron complex molecule |
| Ferrous Sulfate and Vitamin Complex Sustained-release Tablets | Ferrous Sulfate; Vitamin C; Vitamin B |
| Iron Proteinsuccinylate Oral Solution | Iron Proteinsuccinylate |

# Supplementary Table 4. The effect size for different iron formulations

| Iron formulations | N | Effect size (95% CI) | *P1* | I² | *P2* |
| --- | --- | --- | --- | --- | --- |
| RBC (SMD) |  |  |  |  |  |
| Children (subgroup 1) | 7 | -0.66 (-1.06, -0.26) | 0.001 | 88 | 0.101 |
| Ferrous Succinate Tablets | 4 | -0.65 (-1.14, -0.15) | 0.010 | 86 |  |
| Ferrous Sulfate Tablets | 3 | -0.68 (-1.5, 0.14) | 0.103 | 93 |  |
| Pregnant women (subgroup 1) | 5 | 1.23 (-0.21, 2.66) | 0.093 | 97.3 | 0.496 |
| Ferrous Succinate Tablets | 2 | 0.97 (-3.52, 5.47) | 0.671 | 99.2 |  |
| Ferrous Sulfate Tablets | 1 | 1.56 (1.11, 2) | 0.000 |  |  |
| Polysaccharide Iron Complex Capsule | 2 | 1.34 (0.95, 1.72) | 0.000 | 0 |  |
| Pregnant women (subgroup 2) | 13 | 2.53 (2.02, 3.04) | 0.000 | 92.6 | 0.053 |
| Ferrous Succinate Tablets | 7 | 2.77 (2.03, 3.52) | 0.000 | 94.2 |  |
| Iron Dextran Dispersible Tablets | 1 | 0.86 (0.39, 1.32) | 0.000 |  |  |
| Polysaccharide Iron Complex Capsule | 2 | 2.95 (2.05, 3.84) | 0.000 | 81.5 |  |
| Compound Ferrous Sulfate and Folic Acid Tablets | 2 | 2.15 (1.52, 2.78) | 0.000 | 62 |  |
| Iron Proteinsuccinylate Oral Solution | 1 | 2.46 (1.82, 3.1) | 0.000 |  |  |
| Hb (SMD) |  |  |  |  |  |
| Children (subgroup 1) | 7 | 0.16 (-0.13, 0.44) | 0.278 | 78.5 | 0.304 |
| Ferrous Succinate Tablets | 4 | 0.08 (-0.13, 0.3) | 0.448 | 33 |  |
| Ferrous Sulfate Tablets | 3 | 0.31 (-0.38, 0.99) | 0.378 | 91.2 |  |
| Adults (subgroup 1) | 4 | 0 (-0.2, 0.21) | 0.963 | 16.3 | insufficient data |
| Ferrous Succinate Tablets | 1 | 0.11 (-0.19, 0.4) | 0.476 |  |  |
| Ferrous Sulfate Tablets | 1 | -0.34 (-0.96, 0.29) | 0.289 |  |  |
| Ferrous Sulfate and Vitamin Complex Sustained-release Tablets | 2 | -0.02 (-0.4, 0.35) | 0.909 | 49.5 |  |
| Pregnant women (subgroup 1) | 7 | 1.27 (0.21, 2.34) | 0.019 | 96.9 | 0.139 |
| Ferrous Succinate Tablets | 2 | 0.09 (-2.62, 2.79) | 0.949 | 98.5 |  |
| Polysaccharide Iron Complex Capsule | 2 | 1.37 (0.77, 1.98) | 0.000 | 58.4 |  |
| Iron Proteinsuccinylate Oral Solution | 1 | 3.47 (2.7, 4.23) | 0.000 |  |  |
| Compound Ferrous Sulfate and Folic Acid Tablets | 1 | 2.51 (1.92, 3.1) | 0.000 |  |  |
| Compound Ferrous Sufate Granules | 1 | 0.15 (-0.16, 0.47) | 0.337 |  |  |
| Pregnant women (subgroup 2) |  |  |  |  | 0.019 |
| Ferrous Succinate Tablets | 8 | 1.84 (1.45, 2.23) | 0.000 | 84.7 |  |
| Ferrous Sulfate Tablets | 1 | 2.09 (1.6, 2.58) | 0.000 |  |  |
| Polysaccharide Iron Complex Capsule | 2 | 1.86 (0.65, 3.07) | 0.003 | 92.6 |  |
| Compound Ferrous Sulfate and Folic Acid Tablets | 1 | 2.31 (1.75, 2.86) | 0.000 |  |  |
| Iron Proteinsuccinylate Oral Solution | 1 | 1.66 (1.04, 2.29) | 0.000 |  |  |
| Iron Dextran Dispersible Tablets | 1 | 1.79 (1.26, 2.32) | 0.000 |  |  |
| MCV (SMD) |  |  |  |  |  |
| Children (subgroup 1) | 8 | 0.1(-0.02, 0.23) | 0.250 | 0 | 0.337 |
| Ferrous Succinate Tablets | 4 | 0.13 (-0.09, 0.34) | 0.263 | 31.7 |  |
| Ferrous Sulfate Tablets | 4 | 0.1 (-0.07, 0.27) | 0.094 | 0 |  |
| Adults (subgroup 1) | 4 | 0.07 (-0.27, 0.42) | 0.684 | 67.7 | insufficient data |
| Ferrous Succinate Tablets | 1 | -0.21 (-0.5, 0.09) | 0.172 |  |  |
| Ferrous Sulfate Tablets | 1 | -0.26 (-0.89, 0.36) | 0.405 |  |  |
| Ferrous Sulfate and Vitamin Complex Sustained-release Tablets | 2 | 0.33 (-0.04, 0.7) | 0.084 | 47.7 |  |
| Pregnant women (subgroup 1) | 4 | 0.59 (0.19, 0.99) | 0.004 | 66.4 | insufficient data |
| Ferrous Succinate Tablets | 1 | 0.48 (0.04, 0.93) | 0.033 |  |  |
| Ferrous Sulfate Tablets | 1 | 1.14 (0.71, 1.56) | 0.000 | 0 |  |
| Polysaccharide Iron Complex Capsule | 2 | 0.35 (0, 0.7) | 0.047 |  |  |
| Pregnant women (subgroup 2) | 12 | 0.88 (0.68, 1.08) | 0.000 | 64.9 | 0.048 |
| Ferrous Succinate Tablets | 5 | 0.84 (0.49, 1.19) | 0.000 | 77.7 |  |
| Polysaccharide Iron Complex Capsule | 2 | 0.84 (0.48, 1.2) | 0.000 | 41 |  |
| Compound Ferrous Sulfate and Folic Acid Tablets | 2 | 1.22 (0.63, 1.82) | 0.000 | 68.5 |  |
| Iron Proteinsuccinylate Oral Solution | 1 | 1.04 (0.53, 1.55) | 0.000 |  |  |
| Iron Dextran Dispersible Tablets | 1 | 0.46 (0.01, 0.91) | 0.000 |  |  |
| Ferrous Lactate Oral Solution | 1 | 0.81 (0.25, 1.37) | 0.004 |  |  |
| MCH (SMD) |  |  |  |  |  |
| Children (subgroup 1) | 8 | -0.05 (-0.19, 0.1) | 0.529 | 29.1 | 0.98 |
| Ferrous Succinate Tablets | 4 | -0.07 (-0.35, 0.21) | 0.634 | 58.3 |  |
| Ferrous Sulfate Tablets | 4 | 0 (-0.17, 0.17) | 0.988 | 0 |  |
| Adults (subgroup 1) | 4 | -0.15 (-0.34, 0.03) | 0.104 | 0 | insufficient data |
| Ferrous Succinate Tablets | 1 | -0.11 (-0.41, 0.18) | 0.457 |  |  |
| Ferrous Sulfate Tablets | 1 | -0.57 (-1.21, 0.06) | 0.076 |  |  |
| Ferrous Sulfate and Vitamin Complex Sustained-release Tablets | 2 | -0.12 (-0.37, 0.14) | 0.374 | 0 |  |
| Pregnant women (subgroup 1) | 2 | 1.68 (-1.34, 4.71) | 0.280 | 98 |  |
| Ferrous Succinate Tablets | 1 | 3.28 (2.6, 3.95) | 0.000 |  |  |
| Compound Ferrous Sufate Granules | 1 | 0.16 (-0.16, 0.47) | 0.328 |  |  |
| SI (SMD) |  |  |  |  |  |
| Children (subgroup 1) | 5 | 0.03 (-0.12, 0.17) | 0.718 | 0 | 0.99 |
| Ferrous Succinate Tablets | 4 | 0 (-0.17, 0.17) | 0.989 | 0 |  |
| Ferrous Sulfate Tablets | 1 | 0.1 (-0.19, 0.38) | 0.498 |  |  |
| Pregnant women (subgroup 1) | 4 | 1.45 (0.41, 2.48) | 0.006 | 95.1 | insufficient data |
| Ferrous Succinate Tablets | 1 | 2.65 (2.05, 3.26) | 0.000 |  |  |
| Ferrous Sulfate Tablets | 1 | 1.41 (0.97, 1.85) | 0.000 |  |  |
| Polysaccharide Iron Complex Capsule | 1 | 1.6 (1.05, 2.15) | 0.000 |  |  |
| Compound Ferrous Sulfate and Folic Acid Tablets | 1 | 0.2 (-0.11, 0.51) | 0.211 |  |  |
| Pregnant women (subgroup 2) | 12 | 3 (2.27, 3.72) | 0.000 | 95.7 | 0.216 |
| Ferrous Succinate Tablets | 6 | 2.94 (2.12, 3.75) | 0.000 | 94.2 |  |
| Iron Dextran Dispersible Tablets | 1 | 1.74 (1.22, 2.27) | 0.000 |  |  |
| Polysaccharide Iron Complex Capsule | 2 | 2.03 (-0.78, 4.83) | 0.157 | 98.3 |  |
| Compound Ferrous Sulfate and Folic Acid Tablets | 2 | 3.02 (2.28, 3.76) | 0.000 | 62.2 |  |
| Ferrous Lactate Oral Solution | 1 | 7.7 (6.13, 9.27) | 0.000 |  |  |
| SF (SMD) |  |  |  |  |  |
| Pregnant women (subgroup 2) | 2 | 2.54 (1.31, 3.77) | <0.00001 | 92 |  |
| Ferrous Succinate Tablets | 1 | 1.94 (1.57, 2.32) | 0.000 |  |  |
| Polysaccharide Iron Complex Capsule | 1 | 3.22 (2.61, 3.82) | 0.000 |  |  |
| TSAT (SMD) |  |  |  |  |  |
| Pregnant women (subgroup 1) | 2 | 1.66 (0.88, 2.45) | <0.0001 | 77 |  |
| Ferrous Succinate Tablets | 1 | 2.09 (1.54, 2.63) | 0.000 |  |  |
| Polysaccharide Iron Complex Capsule | 1 | 1.28 (0.76, 1.8) | 0.000 |  |  |
| Pregnant women (subgroup 2) | 11 | 2.15 (1.95, 2.35) | 0.000 | 43 | 0.001 |
| Ferrous Succinate Tablets | 5 | 2.3 (2.02, 2.58) | 0.000 | 47.9 |  |
| Polysaccharide Iron Complex Capsule | 2 | 1.95 (1.63, 2.27) | 0.000 | 0 |  |
| Compound Ferrous Sulfate and Folic Acid Tablets | 2 | 2.42 (2.01, 2.82) | 0.000 | 0 |  |
| Iron Dextran Dispersible Tablets | 1 | 1.74 (1.22, 2.27) | 0.000 |  |  |
| Ferrous Lactate Oral Solution | 1 | 1.6 (0.98, 2.21) | 0.000 |  |  |

Note：N, number of trials; *P1*, *P* value for effect size; *P2*, *P* value for meta regression analysis among one population group in terms of the type of iron formulations.
